# The Gluconeogenesis Pathway Is Involved in Maintenance of Enterohaemorrhagic *Escherichia coli* O157:H7 in Bovine Intestinal Content

**DOI:** 10.1371/journal.pone.0098367

**Published:** 2014-06-02

**Authors:** Yolande Bertin, Christiane Deval, Anne de la Foye, Luke Masson, Victor Gannon, Josée Harel, Christine Martin, Mickaël Desvaux, Evelyne Forano

**Affiliations:** 1 Institut National de la Recherche Agronomique, UR454 Microbiologie, Saint-Genès-Champanelle, France; 2 Institut National de la Recherche Agronomique, UMR 1019, Unité de Nutrition Humaine, Centre de Recherche en Nutrition Humaine d’Auvergne, Clermont-Ferrand, France; Clermont Université, Université d’Auvergne, Unité de Nutrition Humaine, BP 10448, Clermont-Ferrand, France; 3 Institut National de la Recherche Agronomique, UMR1213 Herbivores, Plate-Forme d’Exploration du Métabolisme, Saint-Genès-Champanelle, France; 4 Biotechnology Research Institute, National Research Council of Canada, Montreal, Quebec, Canada; 5 Laboratory for Foodborne Zoonoses, Public Health Agency of Canada, Lethbridge, Alberta, Canada; 6 Groupe de Recherche sur les Maladies Infectieuses du Porc, Université de Montréal, Faculté de Médecine Vétérinaire, Saint-Hyacinthe, Québec, Canada; University of Strathclyde, United Kingdom

## Abstract

Enterohaemorrhagic *Escherichia coli* (EHEC) are responsible for outbreaks of food- and water-borne illness. The bovine gastrointestinal tract (GIT) is thought to be the principle reservoir of EHEC. Knowledge of the nutrients essential for EHEC growth and survival in the bovine intestine may help in developing strategies to limit their shedding in bovine faeces thus reducing the risk of human illnesses. To identify specific metabolic pathways induced in the animal GIT, the transcriptome profiles of EHEC O157:H7 EDL933 during incubation in bovine small intestine contents (BSIC) and minimal medium supplemented with glucose were compared. The transcriptome analysis revealed that genes responsible for the assimilation of ethanolamine, urea, agmatine and amino acids (Asp, Thr, Gly, Ser and Trp) were strongly up-regulated suggesting that these compounds are the main nitrogen sources for EHEC in BSIC. A central role for the gluconeogenesis pathway and assimilation of gluconeogenic substrates was also pinpointed in EHEC incubated in BSIC. Our results suggested that three amino acids (Asp, Ser and Trp), glycerol, glycerol 3-phosphate, L-lactate and C4-dicarboxylates are important carbon sources for EHEC in BSIC. The ability to use gluconeogenic substrates as nitrogen sources (amino acids) and/or carbon sources (amino acids, glycerol and lactate) may provide a growth advantage to the bacteria in intestinal fluids. Accordingly, aspartate (2.4 mM), serine (1.9 mM), glycerol (5.8 mM) and lactate (3.6 mM) were present in BSIC and may represent the main gluconeogenic substrates potentially used by EHEC. A double mutant of *E. coli* EDL933 defective for phosphoenolpyruvate synthase (PpsA) and phosphoenolpyruvate carboxykinase (PckA), unable to utilize tricarboxylic acid (TCA) intermediates was constructed. Growth competition experiments between EHEC EDL933 and the isogenic mutant strain in BSIC clearly showed a significant competitive growth advantage of the wild-type strain further illustrating the importance of the gluconeogenesis pathway in maintaining EHEC in the bovine GIT.

## Introduction

Enterohaemorrhagic *Escherichia coli* (EHEC) are Shiga toxin-producing *E. coli* (STEC) responsible for human gastrointestinal illnesses, including bloody diarrhea [1]. These disorders may be complicated by renal dysfunction, including the life-threatening haemolytic-uraemic syndrome (HUS), responsible for acute renal failure in children [2]. Most outbreaks and sporadic cases of bloody diarrhea and HUS have been attributed to EHEC serotype O157:H7. STEC strains have been isolated from the intestine of various healthy domestic and wild animals, but ruminants, mainly cattle, are the principal reservoir [3], [4]. While EHEC strains colonize the bovine gastrointestinal tract, cattle are asymptomatic because they do not express the globotriaosylceramide-3 (Gb3) receptor on their vascular endothelium. Binding to this receptor is thought to be necessary for the pathophysiological effects associated with Shiga toxin in the human host [5]. EHEC strains are transmitted from cattle to humans by means of unpasteurized milk, undercooked meat, fruit, vegetables or water. Hides have been specifically identified as the principal source of EHEC contamination during slaughter [6].

In-depth knowledge of EHEC physiology and metabolism during residence in the bovine intestinal environment is critical to understand how it is shed in animals and to identify nutritional strategies to limit its shedding. According to Freter's nutrient niche theory, intestinal colonization by a given bacterial species requires the consumption of one or a small number of growth-limiting nutrients more efficiently than all of the other competitors in this ecosystem [7]. In the mammalian intestine, nutrients are released from ingested foods and epithelial or bacterial cell debris. The lumen contains a wide variety of nutrients, at various concentrations and many of these are actively absorbed by the host's intestine. In the rumen, growth of *E. coli* O157:H7 is limited or may even be suppressed by the presence of the resident microbiota and by strictly anaerobic conditions [8], [9], [10]. However, *E. coli* O157:H7 may survive passage through the acid barrier of the abomasum, and enter the lumen of the bovine small intestine which likely constitutes a more favourable environment for EHEC growth [11].

To survive and compete, heterotrophic microorganisms have developed the ability to tune their metabolism according to nutrient composition and availability in a given environment [12], [13]. Although glucose is the preferred carbon and energy source for many bacteria, pathogenic isolates are capable of surviving by using a variety of carbon and nitrogen substrates, including other carbohydrates, lipids, glycolipids and amino acids. The capacity of EHEC to adapt to nutrient availability and environmental conditions is key to their residence time and survival in the intestine. However, our knowledge of the nutrients preferentially used by EHEC in the bovine intestine and the metabolic pathways required for persistence and growth is still limited. For example, Snider *et al.* demonstrated that fucose is a critical carbon source for the survival of EHEC in the bovine rectum [14]. Also, *in vivo* colonization experiments showed that the genes *agaB* and *dctA*, coding for the specific transport of N-acetyl-glucosamine (GlcNAc) and C4 dicarboxylic acids respectively, influence EHEC colonization of the bovine gut [15]. More recently, we showed that EHEC could catabolize the monosaccharides, released into the bovine small intestine from the mucus layer covering the enterocytes, simultaneously [16]. Furthermore, using a co-culture model we also demonstrated that mucus-derived carbohydrates (GlcNAc, mannose, N-acetyl neuraminic acid and galactose) confer a competitive growth advantage to EHEC in the bovine small intestine [16]. Similarly, ethanolamine released from phospholipids included in animal, plant and microbial cell membranes is an important nitrogen source that favours EHEC persistence in the bovine small intestine [11].

For their successful survival and multiplication, bacteria must exert specific control over genes required for adaptation to and growth within specific environments. As the transcription of genes encoding catabolic and anabolic reactions can be dynamically altered by bacteria according to nutrient availability, specific metabolic patterns can be deduced from the gene expression profiles [12], [17], [18], [19]. In this study, a whole genome DNA microarray was used for the first time to compare whole transcriptomic profiles of EHEC incubated in bovine small intestine content (BSIC) and minimal medium. The objective of this study was to identify metabolic pathways activated by EHEC in BSIC in order to improve our understanding of the metabolic mechanisms controlling EHEC growth and survival in the bovine gut. Our results highlight the importance of gluconeogenic substrates as carbon and nitrogen sources for EHEC.

## Materials and Methods

### Ethics statement

Animals were slaughtered in accordance with the guidelines of the local Ethics Committee and current INRA ethical guidelines for animal welfare (Permit number: 63345001). BSIC samples were collected after the slaughter of animals required for experiments specifically approved by the “Comité d’éthique en matière d’experimentation animale en Auvergne (Permit number: CE22-08) in the experimental slaughterhouse of the “Unité Mixte de Recherche sur les Herbivores”, INRA, Saint-Genes-Champanelle, France.

### Bacterial strains and growth media

EHEC O157:H7 EDL933 was isolated from contaminated hamburger meat [20]. The spontaneous nalidixic acid-resistant mutant EHEC EDL933 Nal^R^ previously described [16] was used in this study. The BSIC samples were collected as previously described [11], [21], [22]. Briefly, the jejunum and the ileum were removed as a single piece from three beef cattle and the total luminal contents were collected in O_2_-free N_2_-saturated sterile flasks. The BSIC samples were then pooled, rapidly filtered through four layers of cheesecloth and immediately frozen at -80°C until use. Under these conditions of preparation and storage, the microbiota included in BSIC samples remains stable during two months [11]. To obtain sterile BSIC, the intestinal contents were centrifuged twice at 2,000×g for 20 min and the resulting supernatants were filtered through a 0.22-μm nylon filter.

*E. coli* EDL933 was cultured in sterile BSIC or M9 minimal medium supplemented with 0.4% glucose, MgSO_4_ (1 mM), CaCl_2_ (0.1 mM) and trace metals (M9-Glc). Broth cultures were started from a single colony in Luria-Bertani (LB) broth and grown for 8 hours at 37°C with aeration. The bacterial cells were then diluted 50-fold in sterile BSIC or M9-Glc and cultured overnight without aeration (to minimize oxygen availability) at 39°C (bovine temperature). The next day, sterile BSIC or M9-Glc was inoculated by overnight BSIC or M9-Glc broth cultures (1/100 dilution), respectively (15 mL tubes containing 14 mL of BSIC or M9-Glc were used to limit oxygen availability). The cultures were then incubated at 39°C without shaking. The growth rate of *E. coli* EDL933 was very similar in either BSIC or M9-Glc media (μ_max_ 1.25 h^−1^), although the lag phase and growth yield were 2 and 1.5 fold higher in M9-Glc medium, respectively.

### DNA microarray experiments

#### 1) RNA extraction and cDNA labeling

Transcriptome analysis was performed from RNA collected when *E. coli* EDL933 reached the stationary growth phase (4.25 h and 7.5 h in BSIC and M9-Glc respectively). Bacterial suspensions were then centrifuged at 10,000×*g* for 15 min. The supernatants were stored at -20°C for further investigation and the bacterial pellets were rapidly resuspended in two volumes of RNAprotect Bacteria reagent (Qiagen) to stabilize the RNA. The suspensions were then centrifuged at 10,000×*g* for 15 min, and total RNA was purified from the bacterial pellet using the NucleoSpin RNA II kit (Macherey-Nagel). Contaminating DNA was removed using an RNAse-free DNAse I column as described by the manufacturer (Macherey-Nagel). RNA was quantified using a NanoDrop ND-1000 spectrophotometer and RNA integrity was electrophoretically verified by ethidium bromide staining. RNA samples were then precipitated by adding 1/10 volume of 3 M sodium acetate (pH 5.2) and 2.5 volumes of cold 100% ethanol before storage at −80°C until further use.

Total RNA samples (8 µg) were reverse transcribed to Cy3- or Cy5-labeled cDNA using the BD-Atlas PowerScript Fluorescent labeling kit (Invitrogen) and Cy-dye Post-Labelling Reactive Dye Pack (Amersham) following the manufacturers' instructions. Unincorporated Cy-dye was removed using the QIAquick PCR purification kit (Qiagen) and dye incorporation was quantified using a NanoDrop spectrophotometer.

#### 2) Hybridization and scanning

Corning Ultra-Gap II slides (Corning, Acton, MA) were spotted with the MWG *E. coli* O157:H7 array set (Ocimum) at the National Microbiology Laboratory (Canadian Science Centre for Human and Animal Health, Public Health Agency of Canada, Winnipeg). As previously described, the MWG array consists of 6,167 50-mer oligonucleotides covering the genomes of *E. coli* K-12 (MG1655) and *E. coli* O157:H7 strains Sakai (RIMD 0509952) and EDL933 (ATCC 700927) [23]. Description of the array is available at the Gene Expression Omnibus (GEO) database (http://www.ncbi.nlm.nih.gov/projects/geo/) (platform accession number GPL6178). Two arrays were spotted per slide.

Prior to hybridization, the slides were incubated for 50 min at 42°C in prehybridization buffer (5×SSC, 0.1% SDS, 0.1% bovine serum albumin), washed twice at room temperature in distilled water for 10 min and dried by centrifugation for 5 min at 800×*g*. Cy3- and Cy5-labeled cDNA (200 pmol each) were then mixed, dried, resuspended in 50 µL hybridization buffer (5×SSC, 0.1% SDS, 50% deionized formamide, 0.2 g L^−1^ salmon sperm DNA) and denatured for 5 min at 95°C before cooling for 5 min at room temperature. Labelled cDNA was then applied to the microarray slide, covered with Hybri-Slip cover slips (Molecular Probes), placed in hybridization chambers (Corning) and hybridized overnight at 42°C. Following hybridization, the slides were washed twice in 0.1× SSC, 0.1% SDS (42°C, 10 min) and twice in 0.1× SSC (room temperature, 10 min). After drying by centrifugation (5 min at 800×*g*), the hybridized slides were scanned with a SYS-SN-ARRAY Agilent Microarray Scanner (Agilent, Santa Clara, CA, USA) at 10 µm resolution.

#### 3) Data analysis

The signal and background intensity values in both channels for each printed spot were obtained using GenePix Pro 6.0 software (Axon Instruments, USA). Spots with a reference signal lower than background plus two standard deviations were excluded. A total of four arrays were performed using biological replicates (RNA samples were collected from four cultures in BSIC and M9-Glc respectively on separate days). Two microarrays were hybridized with cDNA from BSIC and M9-Glc labelled with cy3 and cy5 respectively and other two hybridizations with cDNA samples conversely labelled. Empty and flagged spots were excluded, and intensities were transformed using a base-2 logarithm. Data normalisation and differential analyses were done using R software. A background correction based on a convolution model which was found to stabilize the variance of the log-ratios (normexp + offset) was processed using limma R package (http://www.bioconductor.org/packages/2.6/bioc/html/limma.html) [24]. The mean spot intensity-dependent dye effect was corrected by performing a global Lowess normalization. The block effect was corrected via subtraction of the median by blocks on each slide following the Anapuce library method [25]. In order to achieve consistency of log-ratio distributions between arrays, the log-ratios of each array were divided by their median-absolute-deviation, a robust measure of dispersion, and multiplied by the mean median-absolute-deviation computed on the four arrays [26]. Technical replicates were then averaged within arrays. Statistical analysis was carried out using the anapuce R package. Differential analysis was done with the Varmixt method which relies on gene expression variances mixture modelling. This method leads to a more powerful test than a t-test assuming a specific variance for each gene, and to a more realistic assumption than assuming a common variance for all genes [27]. The False Discovery Rate was controlled by adjusting p-values using the Benjamini Hochberg method [28]. In this report, only genes showing a >2-fold up-regulation or down-regulation and a Benjamini-Hochberg False Discovery Rate-adjusted *P* value of <0.05 were considered to be differentially regulated.

#### 4) Microarray data accession number

The microarray data have been deposited in NCBI's GEO database (accession number GSE49468).

### In silico analysis

The list of genes was submitted to Database for Annotation, Visualization and Integrated Discovery (DAVID) (resource v6.7) (http://david.abcc.ncifcrf.gov/summary.jsp) to access a relational database of functional annotation [29], [30]. The genes were categorized in Gene Ontology (GO) categories (“Biological Process” or “Compartment cellular”) using the chart feature offered by DAVID (http://www.geneontology.org/GO.nodes.html). The Gene Functional Classification tool in DAVID builds clusters of genes with significantly similar ontologies as tested against whole *E. coli* genomes. For each gene list clustered in the different GO terms, DAVID calculated an “Expression Analysis Systematic Explorer” (EASE) score (also called enrichment score) corresponding to a modified Fisher Exact P-value (ranged from 0 to 1). Fisher Exact P-value = 0 represents perfect enrichment. P-value smaller than 0.05 considered as strongly enriched in the annotation categories was used as standard cut-off level. BLAST (http://www.ncbi.nlm.nih.gov), *x*BASE (http:// *x*base.bham.ac.uk), Kyoto Encyclopedia of Genes and Genomes (KEGG) (www.genome.jp/kegg) and EcoCyc (http://ecocyc.org) servers were also used in this study.

### Quantitative PCR (qPCR)

Quantitative PCR experiments were performed to validate the microarray results. One microgram of each RNA sample was reverse transcribed using the SuperScript II Reverse Transcriptase kit (Invitrogen) with 3 µg of random primer and 100 units of SuperScript II Rnase H. Quantitative PCR runs were carried out using the Mastercycler ep realplex apparatus (Eppendorf) with 20 ng of cDNA, 0.5 µM of each primer, 3 mM of MgCl_2_, 10 µL of SYBR Premix Ex Taq mix (Takara Bio Inc.) in a final volume of 20 µL. Amplification conditions were as follows: 95°C for 15 s, 55°C for 15 s, and 72°C for 20 s. The *tufA* mRNA was used for normalization of mRNA quantification. The relative mRNA quantification was performed using primers designed to specifically amplify fragments of 90 to 200 bp (Table S1). Control samples lacking the reverse transcriptase were included to assess DNA contamination and triplicate samples were amplified in each case. Results were calculated using the comparative cycle threshold method.

### Mutant construction

The sequential construction of the double mutant EDL933Δ*ppsA*Δ*pckA* was performed by using a one-step PCR-based method [31]. The genes *ppsA* (Z2731) and *pckA* (Z4758) were replaced by the genes conferring resistance to chloramphenicol and kanamycin respectively. Primers used to construct the double mutant (ppsA-Cm-F: CGCAGAAATGTGTTTCTCAAACCGTTCATTTATCACAAAAGGATTGTTCGGTGTAGGCTGGAGCTGCTTC, ppsA-Cm-R: TCTTCGGGGATCACATAAACCCGGCGACAAAACGCCGCCGGGGATTTATTCATATGAATATCCTCCTTAGT, pckA-Km-F: CAAAAAGACTTTACTATTCAGGCAATACATATTGGCTAAGGAGCAGTGAAAGCCACGTTGTGTCTCAAAATC and pckA-Km-R: CGTTTTGCTTTCTATAAGATACTGGATAGATATTCTCCAGCTTCAAATCATTAGAAAAACTCATCGAGCA) were designed according to the *E. coli* O157:H7 EDL933 genome sequence. Gene knockouts were confirmed by PCR analysis and DNA sequencing. The wild-type and mutant strains showed similar growth curves when incubated in M9 minimal medium supplemented with glucose (20 mM) for 24 h at 37°C. In contrast to the wild-type strain, the mutant EDL933Δ*ppsA*Δ*pckA* was unable to grow in M9 medium supplemented with sodium succinate (20 mM) as the sole carbon source.

### Bacterial competition experiments

Competition experiments between *E. coli* EDL933 Nal^R^ and the double mutant EDL933Δ*ppsA*Δ*pckA* were performed in BSIC samples containing live endogenous microbiota. Precultures of each *E. coli* strain, inoculated from a single colony, were incubated in LB broth with the appropriate antibiotic for 8 hours at 37°C with aeration. The precultures were then diluted 50-fold in LB broth and grown overnight at 39°C without shaking. The next day, a BSIC sample was inoculated with approximately 5×10^3^ bacteria mL^−1^ of each of the two strains tested and then incubated at 39°C without shaking. At each time point, the co-culture was 10-fold serially diluted in phosphate buffer (PBS) at pH 7.2 and plated on Sorbitol MacConkey (SMAC) agar plates containing nalidixic acid, kanamycin or chloramphenicol (50 µg mL^−1^ each). The plates were then incubated overnight at 37°C and the colony forming units (CFU) were counted. Each experiment was replicated at least three times. The presented values are the log_10_ mean number of CFU mL^−^1 ± standard error. Statistical analysis was done using a Student's t test for paired samples (two-tailed).

### Metabolite quantification

The concentrations of succinate, fumarate, lactate and malate were quantified by NMR as previously described [32]. Briefly, 50 mM TSP-d_4_ was added to 0.5 mL of samples and analyzed from one-dimensional ^1^H NMR spectra. Peak areas were integrated, and the metabolite concentration was calculated relative to TSP-d_4_. Glycerol concentration was measured using an HPLC apparatus as previously described [33]. Briefly, bacterial supernatants (1 mL) were deproteinized with 125 µL of 0.3 M BaOH and 125 µL of 5% ZnSO_4_. After centrifugation, the supernatants were filtered through a 0.22 µm nylon filter and injected in an HPLC apparatus (Agilent 1100 series) fitted with two columns (Rezex ROA 300 × 7.8 nm, Phenomenex) mounted in series in an oven (50 °C) with a refractometer as a detector. The mobile phase consisted of sulphuric acid in ultrapure water pumped at 0.7 mL min^−1^ and 54 bars. Amino acids were quantified by ion-exchange chromatography with post-column ninhydrin detection (Hitachi L8900). The bacterial supernatants were deproteinized as previously described [34] and Norleucine (2.9 mM) was added as the internal standard. Samples were incubated on ice for 15 min and centrifuged at 10000 × *g* for 15 min at 4°C.

## Results and Discussion

### Growth conditions used for performing microarray experiments

Microarray technology was used to identify global gene expression changes in EHEC EDL933 grown in BSIC, relative to the same strain grown in minimal M9 medium supplemented with glucose as the sole carbon source (M9-Glc). As previously reported [11], [16], the growth conditions were designed to mimic the physiological conditions of the bovine gut (see the Materials and Methods section). Transcriptome profiling was performed with RNA samples collected when the bacteria reached the stationary growth phase in BSIC and M9-Glc, respectively. This was done to simulate the growth conditions of the bacterium in its natural environment which are very similar to stationary growth phase conditions during laboratory cultivation [35]. The two bacterial cultures were grown under identical conditions of aeration, pH (≈ 7.2–7.3), temperature and growth phase, and differed only in culture media composition. Indeed, the composition of M9-Glc is completely defined whereas the composition of BSIC is unknown (the small intestine probably contained numerous compounds at low concentration). The aim of the study was to identify metabolic pathways preferentially used by EHEC to assimilate compounds present in BISC and absent in M9-Glc.

### Transcriptomic analysis of EHEC EDL933 growing in BSIC

Statistical analysis identified 658 genes which were transcribed at significantly different levels in EHEC EDL933 grown in BSIC compared to M9-Glc (≥ 2 fold change; *p* value<0.05). Of these, the transcription of 364 and 294 genes were up- and down-regulated, respectively, by the bacteria grown in BSIC compared with those grown in M9-Glc. To validate the microarray results independently, the expression of 34 differentially regulated genes (representing approximately 5% of genes with significantly altered expression) was also measured by qPCR (Table S1). Linear regression calculations showed a significant correlation between qPCR and microarray data (r2 =  0.8038) (Fig. S1).

**Figure 1:**
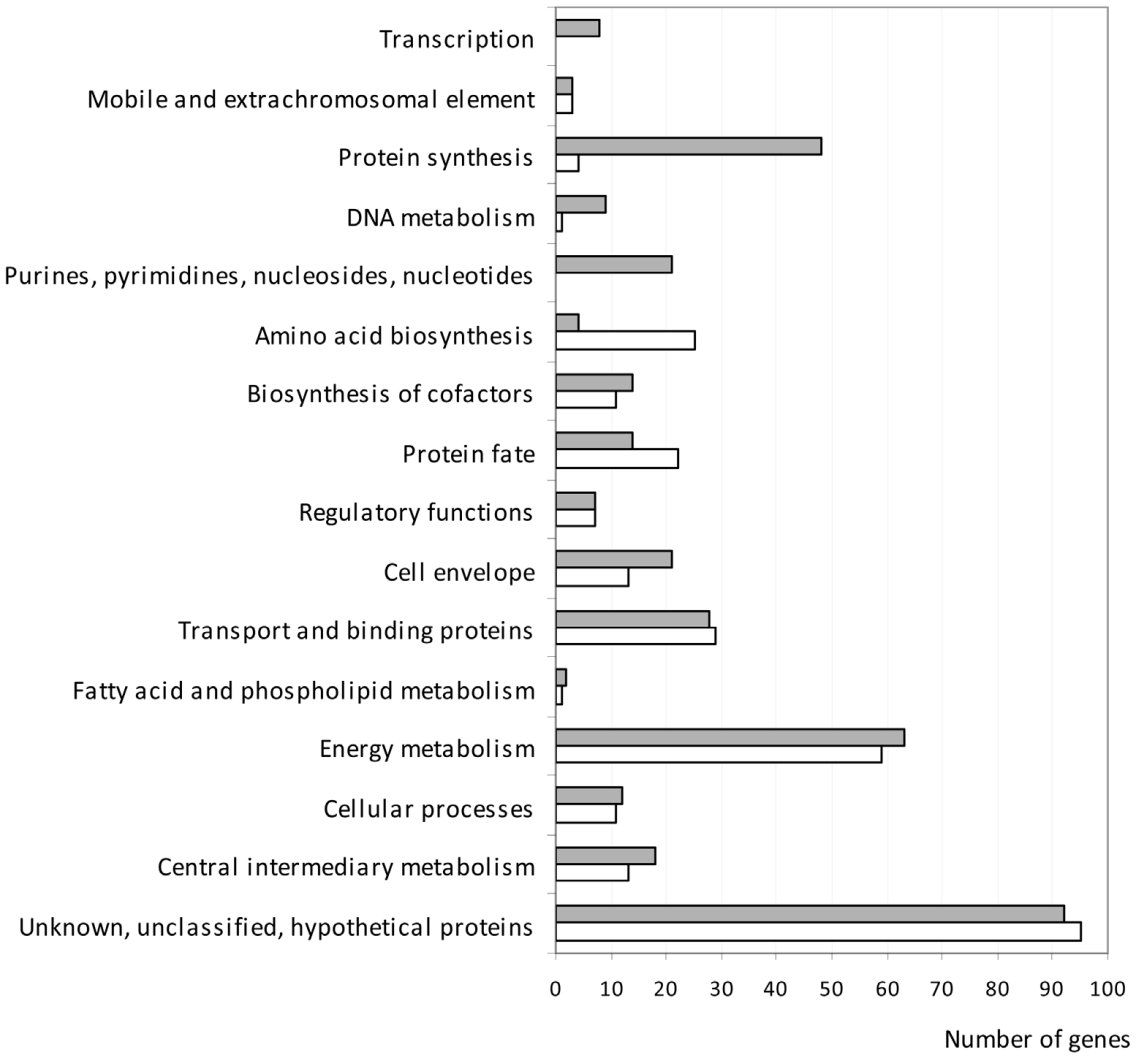
Functional classification of the genes with altered expression in EHEC EDL933 incubated in BSIC compared to M9-Glc. Genes up- and down-regulated in EHEC EDL933 incubated in BSIC compared to M9-Glc are shown in grey and white respectively.

To facilitate subsequent analysis, we first classified the genes into functional groups (Fig. 1). In addition, the differentially expressed genes were classified in different “Gene Ontology” (GO) categories with enrichment scores calculated for each group of genes (see the experimental procedure section and Tables S2 and S3). GO categories and enrichment scores discussed in this study are listed in Table 1. The relative changes in gene expression levels ranged from a 40.6-fold increase for *yidY* (encoding the MdtL multidrug efflux system) to a 25.9-fold decrease for *bioF* (encoding 8-amino-7-oxononanoate synthase) during incubation of *E. coli* EDL933 in BSIC compared with incubation in M9-Glc. Differentially expressed genes were members of nearly all functional categories (Fig. 1). However, a substantial bias in frequency was observed for genes within the “purines, pyrimidines, nucleosides and nucleotide metabolism”, “transcription” and “protein synthesis” functional categories (Fig. 1). Indeed, 77 of the 364 up-regulated genes (21%) in EDL933 incubated in BSIC were found within these three functional groups compared with only 4 of the 294 down-regulated genes (1.4%). Furthermore, genes up-regulated in BSIC were grouped into 11 and 50 catabolic and biosynthetic processes GO categories, respectively (Table S2), whereas genes down-regulated in BSIC were only grouped into 6 and 27 catabolic and biosynthetic processes GO categories, respectively (Table S3). These results are consistent with the notion that *E. coli* EDL933 may need to express more genes encoding catabolic enzymes in order to metabolize the greater variety of substrates available in BSIC compared with M9-Glc.

**Table 1:** Enrichment scores calculated by DAVID for genes up-regulated in EHEC EDL933 incubated in BSIC compared to M9-Glc.

GO category	Gene number	Enrichment score
**Central metabolism and carbon sources**		
Tricarboxylic acid cycle	10	4.1×10^−6^
Gluconeogenesis	5	3.5×10^−3^
Glycerol metabolic process	5	6.9×10^−3^
**Nitrogen sources**		
Amine transport	14	5.0×10^−4^
Amine catabolic process	13	3.0×10^−5^
**Amino acid**		
Amino acid transport	11	8.5×10^−3^
Aromatic amino acid family catabolic process	2	4.3×10^−2^
Glycine catabolic process	3	3.4×10^−2^
Serine family amino acid catabolic process	3	3.4×10^−2^
Tryptophan catabolic process	2	4.3×10^−2^

Since the aim of this report was to increase our understanding of the nutritional basis of EHEC survival in the bovine intestine, we focused on genes up-regulated in EHEC EDL933 incubated in BSIC encoding the transport and metabolism of carbon and nitrogen sources.

### Expression of genes involved in the metabolism of carbon sources

**Figure 2:**
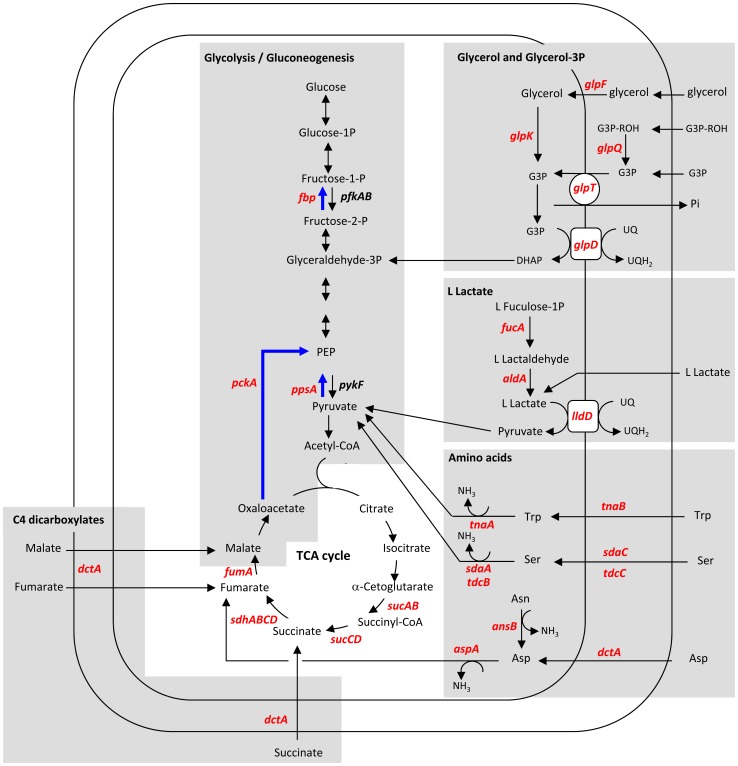
Metabolic pathways involved in the utilization of carbon nutriments by EHEC EDL933 incubated in BSIC. Genes with transcription up-regulated (red) and not altered (black) in EHEC EDL933 incubated in BSIC compared with M9-Glc. Genes encoding enzymes involved in irreversible GNG reactions are shown in blue. G3P: glycerol-3-phosphate; G3P-ROH: glycerophosphodiester; DHAP: dihydroxyacetone phosphate; PEP: phosphoenopyruvate; Pi: inorganic phosphate; UQ: ubiquinone; UQH_2_: ubiquinol.

A high enrichment score was obtained for the genes categorized in the tricarboxylic acid (TCA) cycle GO category when *E. coli* EDL933 was incubated in BSIC in comparison with M9-Glc (Table 1). The genes associated with the TCA cycle are listed in Table 2. The Embden-Meyerof-Parnas (EMP), Entner-Doudoroff (ED) and Pentose-phosphate (PP) pathways are essential for *E. coli* central metabolism and provide the main routes of carbon flux. However, transcription of genes encoding enzymes required for the different steps of the EMP, ED and PP pathways was not significantly altered in EDL933 incubated in BSIC (GEO database: accession number GSE49468). The TCA cycle that can be activated by both glycolytic and gluconeogenic substrates, is essential for respiration-mediated ATP synthesis and the generation of precursors for many other biosynthetic pathways. In this study, the genes classified in the gluconeogenesis (GNG) GO category were significantly up-regulated by EHEC EDL933 in BISC (Table 1). GNG is essentially a reversal of glycolysis but certain GNG steps are irreversible and thus performed by specific enzymes. During incubation in BSIC, EDL933 up-regulated genes involved in irreversible GNG reactions. As shown in Table 2 and Fig. 2, transcription of the genes encoding the irreversible key GNG enzymes, phosphoenolpyruvate (PEP) synthase *(ppsA)*, fructose-1,6-bisphosphatase *(fbp)* and PEP carboxykinase (*pckA)* was induced. In contrast, transcription of the genes coding for the enzymes catalyzing irreversible glycolysis steps (*pfkA, pfkB* and *pykF*) was not significantly altered (GEO database: accession number GSE49468).

**Table 2:** Genes involved in central metabolism and catabolism of gluconeogenic substrates up-regulated in EHEC EDL933 incubated in BSIC compared to M9-Glc.

Gene number	Gene	Function	Fold increase (BSIC vs M9-Glc)	*P* value
**Gluconeogenesis**				
Z5842	*fbp*	Fructose-bisphosphatase	2.01	4.8E-02
Z4758	*pckA*	Phosphoenolpyruvate carboxykinase	4.15	1.8E-03
Z2731	*ppsA*	Phosphoenolpyruvate synthase	2.84	3.6E-03
**TCA cycle**				
Z2615	*fumA*	Fumarate hydratase	2.09	3.3E-02
Z0877	*sdhA*	Succinate dehydrogenase (flavoprotein subunit)	5.35	7.4E-04
Z0878	*sdhB*	Succinate dehydrogenase (iron-sulfur subunit)	3.54	5.7E-03
Z0875	*sdhC*	Succinate dehydrogenase (cytochrome b556)	4.29	6.8E-03
Z0876	*sdhD*	Succinate dehydrogenase (hydrophobic subunit)	5.14	2.5E-03
Z0880	*sucA*	2-oxoglutarate dehydrogenase (decarboxylase component)	3.58	2.3E-03
Z0881	*sucB*	2-oxoglutarate dehydrogenase (E2 component)	2.37	3.6E-03
Z0882	*sucC*	Succinyl-CoA synthetase (b subunit)	3.00	5.6E-03
Z0883	*sucD*	Succinyl-CoA synthetase (a subunit)	2.70	6.8E-03
**Glycerol and glycerol-3P**				
Z4786	*glpD*	Glycerol-3-phosphate dehydrogenase	4.18	7.6E-03
Z5472	*glpF*	Facilitated diffusion of glycerol	3.64	6.0E-03
Z5471	*glpK*	Glycerol kinase	5.74	7.3E-04
Z3497	*glpQ*	Glycerophosphodiester phosphodiesterase	2.38	3.8E-02
Z3498	*glpT*	Glycerol-3-phosphate permease	2.10	5.1E-02
**C4-dicarboxylates**				
Z4942	*dctA*	Aerobic uptake of C4 dicarboxylates and aspartate	5.07	1.4E-03
**L lactate**				
Z5032	*lldD*	L-lactate dehydrogenase	2.34	8.1E-03
Z5031	*lldR*	Transcriptional regulator	2.73	1.4E-02
Z2306	*aldA*	Aldehyde dehydrogenase	2.12	1.9E-02
Z4117	*fucA*	L-fuculose-1-phosphate aldolase	2.86	4.5E-02

In addition to the observed GNG gene up-regulation, EHEC EDL933 induced pathways involved in assimilation of GNG substrates such as glycerol, glycerol-3P, L-lactate and C4-dicarboxylate.

#### 1) Glycerol and glycerol 3-phosphate

A significant enrichment score was obtained for the genes classified in the “glycerol metabolic process” GO category (Table 1). The genes glpF and glpT were up-regulated in EHEC EDL933 during incubation in BSIC (Table 2). These genes encode proteins responsible for the specific transport of glycerol and glycerol 3-phosphate (G3P), respectively, across the inner bacterial membrane (Fig. 2). In addition, the transcription of glpK, glpD and glpQ which encode glycerol kinase, G3P dehydrogenase and phosphodiesterase, respectively, was also induced (Table 2). Under aerobic conditions, GlpK converts glycerol to G3P and then GlpD reduces G3P into dihydroxyacetone phosphate. The latter compound is converted into glyceraldehyde-3P entering the GNG pathway (Fig. 2). GlpQ is required for degrading glycerophosphodiesters into G3P and the corresponding alcohol. In fact, the degradation of glycerol and G3P are closely linked (Fig. 2) and G3P dehydrogenase appears to be an essential membrane enzyme, functioning at the central junction of respiration, glycolysis / GNG and phospholipid biosynthesis.

#### 2) C4-dicarboxylates and L lactate

The gene *dctA* which encode a transporter for C4-dicarboxylates was up-regulated in EHEC EDL933 during incubation in BSIC (Table 2). C4-dicarboxylates (succinate, fumarate and malate) are gluconeogenic substrates that *E. coli* can use as carbon and energy sources [36], [37], [38]. These compounds can directly enter the TCA cycle to participate in the cyclic flow of carbon. Interestingly, *in vivo* colonization experiments showed that *dctA* is required for EHEC colonization of the bovine gut [15].

The genes *lldR* and *lldD* responsible for aerobic L-lactate metabolism were also up-regulated in EHEC EDL933 incubated in BSIC (Table 2). L-lactate dehydrogenase (encoded by *lldD*) is a peripheral membrane protein that catalyzes the oxidation of L-lactate to pyruvate through the respiratory electron transport chain *in vivo* (Fig. 2) and allows *E. coli* to grow in a medium containing L-lactate as the sole carbon source [39]. In addition, the transcription of *aldA* and *fucA* encoding aldehyde dehydrogenase and L-fuculose-1-phosphate aldolase, respectively, was induced in EHEC EDL933 incubated in BSIC. These genes are required for the conversion of L-fuculose-phosphate and L-lactaldehyde to L-lactate (Fig. 2; Table 2).

### Expression of genes involved in the metabolism of nitrogen sources

In this report, genes in the “amine transport” and “amine catabolic process” GO categories were significantly up-regulated in EHEC EDL933 during incubation in BSIC (Table 1). Induction of these genes is likely indicative of nitrogen mobilization in EHEC to support the biosynthesis of compounds essential for bacterial growth such as peptides, proteins and nucleosides. In-depth microarray results analysis showed that the genes encoding the transport and / or catabolism of ethanolamine, urea and agmatine were up-regulated during incubation of EDL933 in BSIC.

#### 1) Ethanolamine

**Figure 3:**
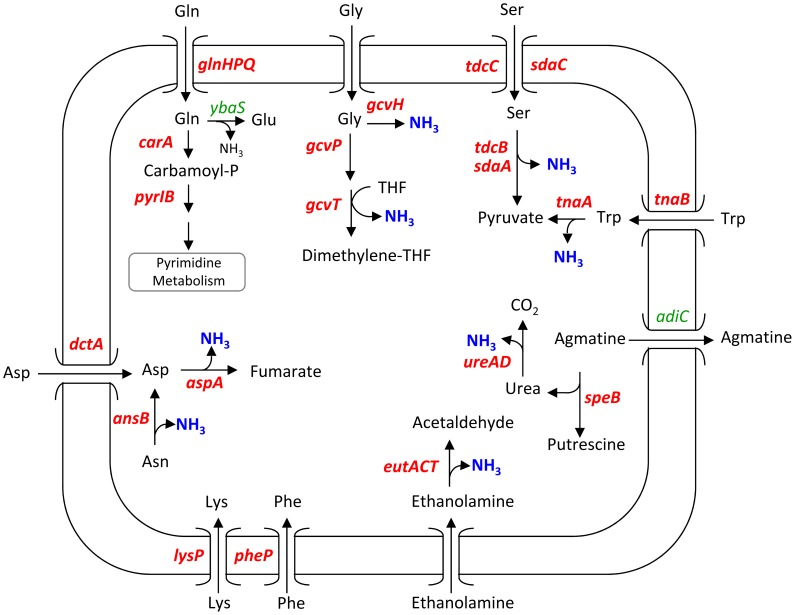
Metabolic pathways involved in the release of ammonia by EHEC EDL933 incubated in BSIC. Genes up- and down-regulated in EHEC EDL933 incubated in BSIC compared to M9-Glc are shown in red and green respectively. The release of ammonia is indicated in blue. THF: tetrahydrofolate.

Fourteen genes in the *eut* operon, which encodes the catabolism of ethanolamine (EA), were up-regulated in EHEC EDL933 incubated in BSIC (Table 3). In particular, the transcription of *eutACT* encoding ethanolamine ammonia-lyase (enzyme converting ethanolamine to acetaldehyde and free ammonia) was induced (Fig. 3). The increased expression of *eut* transcripts during incubation of EDL933 in BSIC observed in this report was previously reported based on qPCR results [11]. Indeed, we have shown that free EA: i) constitutes an important nitrogen source for EHEC in BSIC and ii) is responsible for the induction of the *eut* gene cluster [11]. EA is part of phosphatidylethanolamine, the most abundant phospholipid in bacterial, animal and plant cells membranes and is present in BSIC samples [11]. Consequently, EA constitutes a constantly renewed source of nitrogen in the mammalian intestine and the ability to metabolize this compound likely favours EHEC persistence in this ecological niche [11].

**Table 3:** Genes involved in the transport and utilization of nitrogen sources up-regulated in EHEC EDL933 incubated in BSIC compared to M9-Glc.

Gene number	Gene	Function	Fold change (BSIC vs M9-Glc)	*P* value
**Ethanolamine**				
Z3707	*eutA*	Reactivating factor for ethanolamine ammonia lyase	2.28	1.4E-02
Z3705	*eutC*	Ethanolamine ammonia lyase subunit	2.59	1.8E-03
Z3711	*eutE*	Aldehyde dehydrogenase in ethanolamine utilization	3.65	3.0E-04
Z3709	*eutG*	Alcohol dehydrogenase in ethanolamine utilization	2.04	8.8E-03
Z3714	*eutI*	Ethanolamine utilization	8.07	6.8E-07
Z3710	*eutJ*	chaperonin in ethanolamine utilization	2.76	3.0E-03
Z3703	*eutK*	Carboxysome structural protein (ethanolamine utilization)	2.24	2.5E-03
Z3704	*eutL*	Carboxysome structural protein (ethanolamine utilization)	2.17	4.8E-03
Z3713	*eutM*	Carboxysome structural protein (ethanolamine utilization)	17.11	5.6E-09
Z3712	*eutN*	Carboxysome structural protein (ethanolamine utilization)	9.42	2.9E-07
Z3717	*eutP*	Carboxysome structural protein (ethanolamine utilization)	9.03	4.9E-06
Z3716	*eutQ*	Ethanolamine utilization (unknown function)	8.67	1.4E-05
Z3718	*eutS*	Carboxysome structural protein (ethanolamine utilization)	4.97	6.9E-04
Z3715	*eutT*	Cobalamin adenosyltransferase (ethanolamine ammonia lyase)	9.54	4.6E-06
**Urea**				
Z4281	*speB*	Agmatinase	2.14	3.1E-03
Z1582	*ureA*	Urease structural subunit A	3.21	6.1E-04
Z1583	*ureB*	Urease b subunit	2.38	2.5E-03
Z1581	*ureD*	Urease accessory protein D	3.36	1.7E-03

#### 2) Urea and agmatine

The genes *ureABD* were up-regulated in EHEC incubated in BSIC (Table 3). The *ure* genes encode the enzyme urease that catalyzes the hydrolysis of urea into one carbon dioxide and two ammonium molecules (Fig. 3). Interestingly, the presence of the *ure* gene cluster appears to confer a competitive colonization advantage to STEC strains in the mouse intestine [40]. The authors suggested that urease provides the bacterial cell with an easily assimilated source of nitrogen to gain a metabolic advantage over other endogenous microbiota [40]. Despite the presence of the *ure* gene cluster, EHEC EDL933 displays urease-negative phenotype *in vitro* when cultured on Christensen urea agar plates [41], [42]. However, *ure* genes are regulated by Fur (ferric uptake regulator) or an unidentified *trans*-acting factor and it has been postulate that the pathogen is capable of producing functional urease in the intestine given the correct physiological conditions [41]. The *in vivo* production of urease by EHEC has not been documented, and the conditions necessary to induce these *ure* genes in the bovine intestine are unknown.

The gene *speB* encoding an agmatine ureohydrolase that catalyzes the release of putrescine and urea from agmatine was also up-regulated during incubation in BSIC (Table 3), whereas the transcription of *adiC* encoding the transport of agmatine out of the cell was repressed (9.26-fold decrease in expression; *P:* 0.0000104) (Fig. 3). The lumen of the mammalian gut contains a high amount of agmatine which is released from i) the gut microflora, ii) desquamated gastrointestinal epithelial cells and iii) ingested food [43]. This compound could be used by EHEC to generate ammonia in the bacterial cytoplasm and act as a source of nitrogen. Furthermore, in *E. coli*, arginine decarboxylase catalyzes agmatine formation by the decarboxylation of arginine as the first step of polyamine biosynthesis (that play an important role in the stabilization of DNA and the modulation of genes translation) [44].

Further studies are needed to investigate urease production by EHEC in the animal digestive tract, as well as agmatine metabolism by this pathogen.

### Expression of genes involved in the metabolism of amino acids

A significant enrichment score was calculated for the genes categorized in the “amino acid transport” GO category (Table 1). The genes encoding the transporters for aspartate *(dctA)*, serine (*sdaC, tdcC*), tryptophan (*tnaB*), lysine (*lysP*) and phenylalanine (*pheP*) into the bacterial cells were up-regulated in EHEC EDL933 incubated in BSIC (Table 4, Fig. 2 and 3).

**Table 4:** Genes involved in the transport and catabolism of amino acids up-regulated in EHEC EDL933 incubated in BSIC compared to M9-Glc.

Gene number	Gene	Enzyme or function	Fold increase (BSIC vs M9-Glc)	*P* value
**Carbon and nitrogen sources**				
Z4302	*ansB*	L-asparaginase II	4.48	6.9E-04
Z5744	*aspA*	Aspartate ammonia-lyase	4.62	3.6E-03
Z0037	*carA*	Carbamoyl-phosphate synthetase	4.84	1.1E-03
Z4942	*dctA*	Aerobic uptake of C4-dicarboxylic acids and aspartate	5.07	1.4E-03
Z5856	*pyrB*	Aspartate carbamoyltransferase (catalytic subunit)	2.78	4.6E-03
Z5855	*pyrI*	Aspartate carbamoyltransferase (regulatory subunit)	2.14	2.1E-02
Z2857	*sdaA*	L-serine deaminase	2.93	2.5E-03
Z4113	*sdaC*	Serine uptake	3.09	1.1E-03
Z4468	*tdcC*	Anaerobically import of threonine and serine	3.90	1.8E-03
Z5203	*tnaA*	Tryptophanase	23.41	3.1E-10
Z5204	*tnaB*	Low affinity tryptophan permease	3.60	1.2E-02
**Nitrogen sources**				
Z4241	*gcvH*	Glycine cleavage system	9.43	4.2E-07
Z4240	*gcvP*	Glycine decarboxylase of glycine cleavage system	4.36	4.8E-04
Z3738	*gcvR*	Transcriptional regulation of gcv operon	2.63	1.6E-03
Z4242	*gcvT*	Aminomethyltransferase of glycine cleavage system	17.37	2.9E-09
Z1033	*glnH*	Component of glutamine high-affinity transport system	5.66	1.3E-04
Z1032	*glnP*	Component of glutamine high-affinity transport system	11.34	2.3E-06
Z1031	*glnQ*	Component of glutamine high-affinity transport system	8.47	4.6E-05
Z3413	*lysP*	Lysine-specific permease	2.11	7.2E-03
Z0715	*pheP*	Phenylalanine-specific transport system	2.20	2.8E-03

Gluconeogenic amino acids are both carbon and nitrogen sources for many bacteria: ammonia is first release due to deamination and second, deaminated amino acids yield α-keto acids that can enter either directly or indirectly into the TCA cycle. The transcription of the genes encoding the degradation of gluconeogenic amino acids such as serine, tryptophan and aspartate was induced during incubation of EHEC EDL933 in BSIC. The genes encoding serine deaminase (*sdaA*) and tryptophanase (*tnaA*) converting serine and tryptophan to pyruvate, respectively, and aspartate ammonia-lyase (*aspA*) required to degrade aspartate to fumarate, were also up-regulated (Table 4, Fig. 2 and 3). In addition, the transcription of *ansB*, which encodes asparaginase II, an enzyme that catalyzes the conversion of asparagine to aspartate, was induced (Table 4, Fig. 2 and 3).

The genes *gcvHPRT* coding for the glycine cleavage system which catalyzes the release of ammonia from glycine, were up-regulated (Table 4; Fig. 3) while the transcription of *glyA*, encoding serine hydroxymethyltransferase required to convert 5,10-methylene-THF to serine, was not altered. This suggested that glycine could only be used as nitrogen source by EHEC during incubation in BSIC.

Genes that code for the specific transport of glutamine across the bacterial membrane (*glnHPQ*) were up-regulated (Table 4; Fig. 3), whereas *ybaS*, which encodes glutaminase (converting glutamine to glutamate and ammonia), was down-regulated in EHEC EDL933 incubated in BSIC (6.9-fold decrease; *P*: 0.00003) (Fig. 3). In contrast, the EHEC strain induced the transcription of *carA* encoding carbamoyl-phosphate synthetase as well as *pyrB and pyrI* encoding aspartate carbamoyltransferase required to direct the flux of glutamine to pyrimidine synthesis (Fig. 3).

As shown above, genes coding for the transport of lysine and phenylalanine were up-regulated in EHEC EDL933, but the significance of this is unclear as i) phenylalanine cannot be used as a carbon or nitrogen source by *E. coli* [45] and ii) lysine is a ketogenic amino acid ultimately degraded to carbon dioxide in the TCA cycle. However, the induction of genes encoding specific transporters suggests that phenylalanine and lysine could be imported into the bacterial cytoplasm to serve as building blocks during protein synthesis.

### Role of gluconeogenesis during the growth of EHEC EDL933 in BSIC

From our transcriptomic analyses, a central role for the GNG pathway was pinpointed in *E. coli* EDL933 incubated in BSIC suggesting that the utilization of gluconeogenic substrates provides a growth advantage for the bacteria. It is well documented that PEP synthase (PpsA) (converting pyruvate to PEP) and PEP carboxykinase (PckA) (converting oxaloacetate to PEP) are two key enzymes in the GNG pathway [46], [47], [48]. To perform growth competition experiments, we constructed the double mutant EDL933Δ*ppsA*Δ*pckA* to completely block the conversion of gluconeogenic substrates and TCA cycle intermediates to PEP. A similar double mutant has been previously used to study the role of the GNG pathway in the virulence of *Salmonella enterica* in mice and the colonization of the mouse intestine by EHEC [46], [47]. The wild-type and mutant strains used in our study showed similar growth curves when incubated in M9 minimal medium supplemented with glucose (20 mM) as the sole carbon source. However, in contrast to the wild-type strain, EDL933Δ*ppsA*Δ*pckA* was unable to grow in M9 medium supplemented with sodium succinate (20 mM) as the sole carbon source.

**Figure 4:**
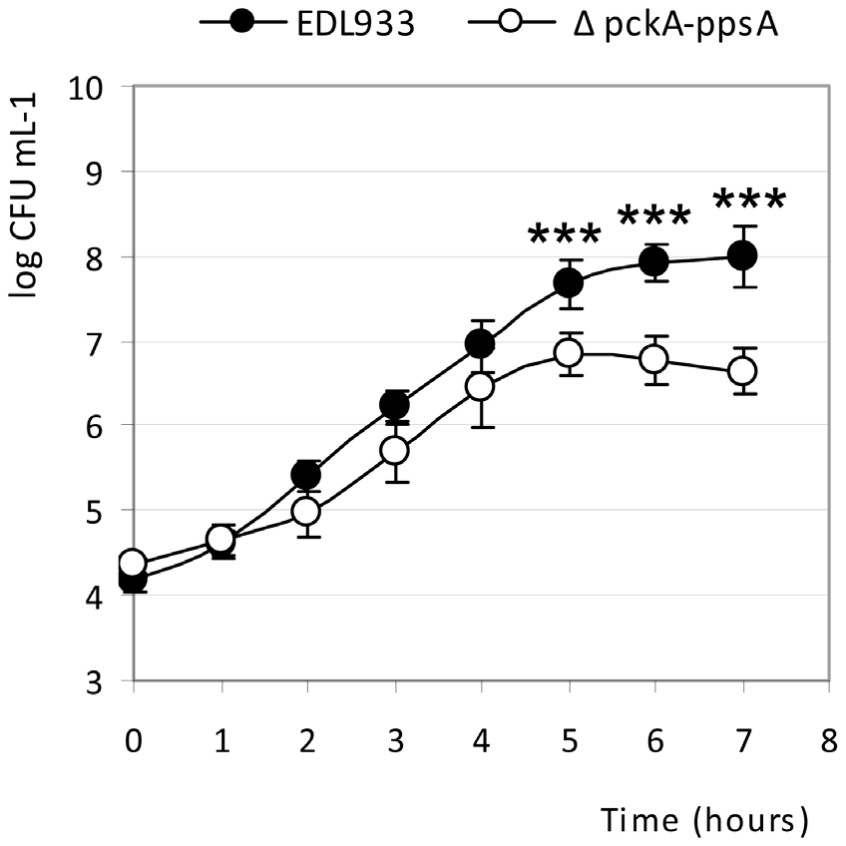
Growth competition assays between EHEC EDL933 and its mutant EDL933 Δ*ppsA*Δ*pckA*. The BSIC samples were inoculated with a 1:1 mixture of the two strains. Bars represent the SEM of three independent experiments. ***, denotes statistical significance, P <0.01 as determined by the Student *t* test for paired samples.

Growth competition assays are usually performed to compare the growth pattern of a wild-type strain and its isogenic mutant co-incubated in biological fluids or liquid growth medium [11], [49], [50], [51]. A mutant strain that does not compete efficiently for nutrients fails to reach the same population density as the parent strain, whereas similar growth curves indicate that both strains are able to use limiting nutrients equally well or do not compete for the same limiting nutrient. The *E. coli* strains EDL933 and EDL933Δ*ppsA*Δ*pckA* were co-incubated in BSIC samples containing live endogenous microbiota under growth conditions that mimic the physiological conditions in BSIC (see the experimental procedure section). As shown in Fig. 4, similar growth curves were observed during the first 4 h of co-incubation, suggesting that nutrients present in BSIC were used by the two strains with equal efficiency. However, after 5 h of co-incubation, a significant growth defect was observed for EDL933Δ*ppsA*Δ*pckA* compared with the wild type strain. These results demonstrated that activation of the GNG pathway confers a competitive growth advantage to EHEC EDL933 in BSIC and suggested that assimilation of gluconeogenic substrates is required for maximal growth of EHEC in the bovine small intestine.

To analyze potential assimilation of gluconeogenic substrates by EHEC EDL933, BSIC was then analyzed by NMR to measure succinate, fumarate, and lactate levels and by HPLC for glycerol and amino acid quantification. A total of 45 mM of free amino acids was detected, including aspartate (2.4 mM) and serine (1.9 mM). Although the transcription of the genes encoding proteins responsible for the transport and the catabolism of tryptophan was induced in EDL933 cultured in BSIC, tryptophan was below the limit of detection (15 µM). Glycerol (5.8 mM) and lactate (3.6 mM) were also detected in BSIC while succinate and fumarate were at levels under the limit of detection. Taken together, the results showed that aspartate, serine, glycerol and lactate are released into the bovine intestine and may constitute important gluconeogenic substrates that can be used by EHEC.

## Conclusion

Since environmental conditions direct the expression of suites of genes necessary for optimal bacterial growth, our data indicate that the utilization of gluconeogenic substrates may play an important role in the colonization of the bovine intestine by EHEC. Gluconeogenic substrates appear to be nitrogen sources (amino acids) and carbon sources (amino acids, glycerol, L-lactate). Accordingly, it is well documented that the GNG pathway contributes to bacterial fitness *in vivo* and is required by pathogenic *Enterobacteriaceae* during host infection [46], [47], [52], [53]. In particular, utilization of gluconeogenic substrates is required for a maximal colonization of the mouse intestine by EHEC [47].

Significant levels of gluconeogenic substrates are likely present in the bovine intestinal environment. Lactate-producing bacteria such as *Lactobacillus, Streptococcus* or *Succinivibrio* sp. are part of the bovine intestinal microbiota and produce lactate (from carbohydrate fermentation) that can accumulate in the bovine intestine [54]. Aspartate and serine are abundant amino acids present in the mucus of the bovine small intestine [55] and may constitute direct sources of nutrients from the intestinal epithelium. Serine is also part of phosphatidylserine (a phospholipid present in the leaflet of prokaryote and eukaryote cell membranes) and may be released into BSIC from intestinal epithelium and cell debris from the endogenous microbiota. Glycerol is part of phospholipids (phosphatidylethanolamine, phosphatidylserine and phosphatidylcholine) present in cell membranes and, like serine, is released into the BSIC during cell renewal. It is interesting to note that glycerol is used as a dietary supplement for cattle as i) a substitute for starch-based ingredients (such as corn), ii) energy supplementation, and iii) a preventive aid for metabolic problems (such as ketosis) [56], [57]. Glycerol is inexpensive and is currently abundant because it is a by-product of biodiesel fuel production. Consequently there is a renewed interest in incorporating this feedstock into ruminant diets. Interestingly, only 80% of the glycerol added to cattle diets is metabolized anaerobically by the ruminal microbiota [58], suggesting that part of the dietary glycerol can reach the small intestine.

The genome-wide expression profiling described in this report highlights the capacity of EHEC to adapt to the bovine digestive environment and opens numerous new avenues for future investigations. In particular, defining the precise role of different gluconeogenic substrates during the growth of EHEC in the bovine intestine should enhance our understanding of their role in the physiology of these pathogens, and may assist us in discovering new nutritional or ecological strategies (for example the use of probiotics) to limit EHEC carriage by ruminants.

## Supporting Information

Fig. S1 Comparison of fold-changes of genes expression obtained by microarray and q-PCR. Thirty four genes with significantly altered expression in EHEC EDL933 cultured in BSIC compared to M9-Glc (both up and down-regulated) were selected from microarray data. The gene list is shown in Table S1. The expression ratios obtained by q-PCR and log2-transformed fold changes were plotted against one another. Linear regression calculations showed a significant correlation between qPCR and microarray data (r2 =  0.8038).
(TIF)

Table S1 Sequence of primers used in relative mRNA quantification.
(DOC)

Table S2 Classification of genes up-regulated in EHEC EDL933 incubated in BSIC compared with cells incubated in M9-Glc. The genes were classified in different “Gene Ontology” (GO) categories with enrichment scores calculated for each genes group.
(DOC)

Table S3 Classification of genes down-regulated in EHEC EDL933 incubated in BSIC compared with cells incubated in M9-Glc. The genes were classified in different “Gene Ontology” (GO) categories with enrichment scores calculated for each genes group.
(DOC)
