# Supplementary figures and images for: The Gluconeogenesis Pathway Is Involved in Maintenance of Enterohaemorrhagic Escherichia coli O157:H7 in Bovine Intestinal Content
Source: PLoS One. 2014 Jun 2;9(6):e98367. doi: 10.1371/journal.pone.0098367 (PMC4041753; doi:10.1371/journal.pone.0098367)

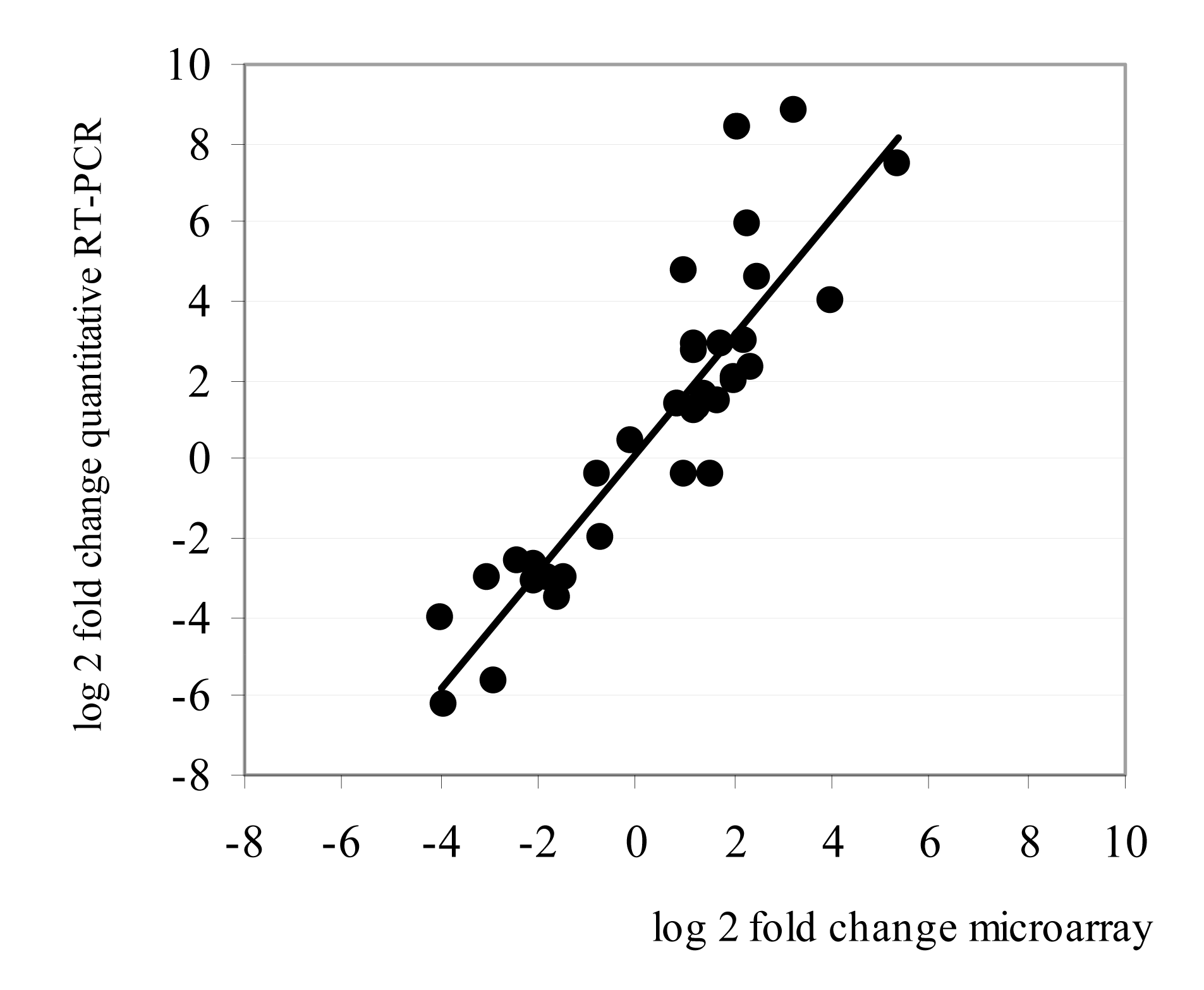

Supplement: Fig. S1 — Comparison of fold-changes of genes expression obtained by microarray and q-PCR. Thirty four genes with significantly altered expression in EHEC EDL933 cultured in BSIC compared to M9-Glc (both up and down-regulated) were selected from microarray data. The gene list is shown in Table S1. The expression ratios obtained by q-PCR and log2-transformed fold changes were plotted against one another. Linear regression calculations showed a significant correlation between qPCR and microarray data (r2 = 0.8038). (TIF) [file pone.0098367.s001.tif]
