# Supplementary material for: The Gluconeogenesis Pathway Is Involved in Maintenance of Enterohaemorrhagic Escherichia coli O157:H7 in Bovine Intestinal Content
Source: PLoS One. 2014 Jun 2;9(6):e98367. doi: 10.1371/journal.pone.0098367 (PMC4041753; doi:10.1371/journal.pone.0098367)
Supplement: Table S1 — Sequence of primers used in relative mRNA quantification. (DOC) [file pone.0098367.s002.doc]

| **Table S1** Sequence of primers used in relative mRNA quantification. | | | |  |
| --- | --- | --- | --- | --- |
|  |  |  |  |  |
| Gene | Name | Forward | Reverse | Reference |
|  | number |  |  |  |
| *acrA* | Z0578 | ATTAGCGGTCGCATTGGTAA | ATCAGCGACACTTTGGCTTT | This study |
| *adhE* | Z2016 | CTGGCAGGCTTCTCTGTACC | TGCTTTTTCTACCGCGTCTT | This study |
| *aspA* | Z5744 | CTTGCTCTCTTCTGGTCCA | AACATAGCCTGGCCAATGAC | This study |
| *bioB* | Z0994 | AGACTCAGGCGATGTGCTTT | AGACTCAGGCGATGTGCTTT | This study |
| *bioC* | Z0996 | GCAACACGCAGAGTTACAGC | TGGTCTGCAGCATCTTTCTG | This study |
| *dctA* | Z4942 | AGTTCTTCACGGATGTAGC | CATGCGTCTGGCACCTATTG | This study |
| *dnaK* | Z0014 | TGTTCAGGGTGGTGTTCTGA | GATGGTTACCGCAGACTGGT | This study |
| *espA* | Z5107 | CGGCACAAAAGATGGCTAAT | GCACATCAGAACGTGCACTC | This study |
| *eutA* | Z3707 | ATGCCCAGAGCTTTACCAAA | GCTGGATCTTGATCCCAAAA | [16] |
| *eutD* | Z3714 | GTTTTTCCGGATGCGTTAGA | CCAGCGATGAGCAAATTCTT | [16] |
| *fbp* | Z5842 | ATGCAAAATACGTGGTGCTG | AGCATGGTAGAGGAGCCGTA | This study |
| *fucA* | Z4117 | AGGATGGGATGCTGATTACG | CGGTCGGTTAAGAATGGAAA | [16] |
| *gadE* | Z4925 | TGCCCCATAAGAATTCACAA | GTGATACCCAGGGTGACGAT | This study |
| *galK* | Z0927 | AGTTCTTCCGCTTCACTGGA | GAGCGACAGTCAATCAGCAA | [16] |
| *glgS* | Z4401 | CGATTTCCTAGCGCGTAGTT | CAGTGTTCTAATTCAAGTTCTC | This study |
| *glpD* | Z4786 | TTGGCGCAAATTCAGTGT | TCGATATCTTCCGCTTCCAC | This study |
| *hisC* | Z3183 | CGAAAGCGGTGATTGAAAAT | CTGTACATGCCGTACGTTGG | This study |
| *hycB* | Z4032 | CGCTGGATATTCCTGCAAAT | CCAGATGCAGGGCTTTAGTC | This study |
| *ldhA* | Z2329 | CTTTCACCGCATCTGCAATA | CTTCGATCCGTCCATCTCAT | This study |
| *lldD* | Z5032 | GATGCTGCGCATTCTGAAAG | ACTGGTATTGACGATCATCAC | This study |
| *manA* | Z2616 | CTGTTCTCTCCGCTGTTGCT | TGGCTTCGAACTTCACATTG | [16] |
| *marA* | Z2170 | TCAGAGCGTTCGGGTTACTC | TGAAGGTTCGGGTCAGAGTT | This study |
| *mdtI* | Z2593 | CTGCCTTTAGTGCGCTTTCT | TCAGGCAAGTTTCACCATGA | This study |
| *mdtL* | Z5205 | CCGGTATTGCTGATGGAAAT | TGGGAAGGTGAAACGGTAAG | This study |
| *pckA* | Z4758 | GTACGCAGAAGTGCTGGTGA | GGCAGAGTGAAGGTTTCTGC | This study |
| *pfkA* | Z5460 | GAAAGAAACCGGTCGTGAAA | TTTCGATAGCGTCGATGATG | This study |
| *poxB* | Z1105 | GTTAAAACCTGCGCCAGAAG | GGGCATGAACAATAGGTGCT | This study |
| *pykF* | Z2704 | TGCTGGATTCCATGATCAAA | AACTCGAGACGGCTGTTCAT | This study |
| *sfaA* | Z1538 | CGGTGCAACTTTAGCTGATG | CCAGAAGTGCTGGAATCCAT | This study |
| *speD* | Z0130 | GCGCCGATATTGAAGTCTCT | CGCCTTCATATCGTCAGACA | This study |
| *speE* | Z0131 | AACCAGCCAGACCTTTGATG | TAATGGCTGAGTTTGCGATG | This study |
| *stx2B* | Z1465 | TATATCAGTGCCCGGTGTGA | CATTATTAAACTGCACTTCAGC | This study |
| *tdcD* | Z4467 | GGAAGGCTTGATGATGGGTA | GCAATTCGGTGAACAAAGGT | This study |
| *Z2200* | Z2200 | GGTCAGGTTCGTTCTTCTC | GAATACCGACATTTTGGGCAG | This study |
