# Supplementary material for: The Gluconeogenesis Pathway Is Involved in Maintenance of Enterohaemorrhagic Escherichia coli O157:H7 in Bovine Intestinal Content
Source: PLoS One. 2014 Jun 2;9(6):e98367. doi: 10.1371/journal.pone.0098367 (PMC4041753; doi:10.1371/journal.pone.0098367)
Supplement: Table S2 — Classification of genes up-regulated in EHEC EDL933 incubated in BSIC compared with cells incubated in M9-Glc. The genes were classified in different “Gene Ontology” (GO) categories with enrichment scores calculated for each genes group. (DOC) [file pone.0098367.s003.doc]

| **Table S2 Classification of genes up-regulated in EHEC EDL933 incubated in BSIC compared with cells incubated in M9-Glc** | | |
| --- | --- | --- |
|  |  |  |
| GO category | Count | Enrichment score |
| [translation](http://www.ebi.ac.uk/ego/DisplayGoTerm?id=GO:0006412) | 40 | 8.90E-29 |
| [nitrogen compound biosynthetic process](http://www.ebi.ac.uk/ego/DisplayGoTerm?id=GO:0044271) | 62 | 4.50E-19 |
| [nucleobase, nucleoside and nucleotide interconversion](http://www.ebi.ac.uk/ego/DisplayGoTerm?id=GO:0015949) | 17 | 2.80E-16 |
| [nucleotide biosynthetic process](http://www.ebi.ac.uk/ego/DisplayGoTerm?id=GO:0009165) | 24 | 5.00E-12 |
| [ribonucleotide biosynthetic process](http://www.ebi.ac.uk/ego/DisplayGoTerm?id=GO:0009260) | 17 | 3.10E-10 |
| [ribonucleotide metabolic process](http://www.ebi.ac.uk/ego/DisplayGoTerm?id=GO:0009259) | 17 | 3.80E-10 |
| [aerobic respiration](http://www.ebi.ac.uk/ego/DisplayGoTerm?id=GO:0009060) | 15 | 1.00E-08 |
| [purine ribonucleotide biosynthetic process](http://www.ebi.ac.uk/ego/DisplayGoTerm?id=GO:0009152) | 14 | 3.00E-08 |
| [purine ribonucleotide metabolic process](http://www.ebi.ac.uk/ego/DisplayGoTerm?id=GO:0009150) | 14 | 3.60E-08 |
| [ncRNA metabolic process](http://www.ebi.ac.uk/ego/DisplayGoTerm?id=GO:0034660) | 23 | 3.70E-08 |
| [purine nucleotide biosynthetic process](http://www.ebi.ac.uk/ego/DisplayGoTerm?id=GO:0006164) | 15 | 8.20E-08 |
| [purine nucleotide metabolic process](http://www.ebi.ac.uk/ego/DisplayGoTerm?id=GO:0006163) | 15 | 1.30E-07 |
| [generation of precursor metabolites and energy](http://www.ebi.ac.uk/ego/DisplayGoTerm?id=GO:0006091) | 32 | 1.50E-07 |
| [energy derivation by oxidation of organic compounds](http://www.ebi.ac.uk/ego/DisplayGoTerm?id=GO:0015980) | 23 | 2.30E-07 |
| [glutamine metabolic process](http://www.ebi.ac.uk/ego/DisplayGoTerm?id=GO:0006541) | 10 | 2.70E-07 |
| [tRNA metabolic process](http://www.ebi.ac.uk/ego/DisplayGoTerm?id=GO:0006399) | 18 | 4.20E-07 |
| [pyrimidine nucleotide metabolic process](http://www.ebi.ac.uk/ego/DisplayGoTerm?id=GO:0006220) | 9 | 5.80E-07 |
| [cofactor catabolic process](http://www.ebi.ac.uk/ego/DisplayGoTerm?id=GO:0051187) | 11 | 8.40E-07 |
| [coenzyme catabolic process](http://www.ebi.ac.uk/ego/DisplayGoTerm?id=GO:0009109) | 11 | 8.40E-07 |
| [glutamine biosynthetic process](http://www.ebi.ac.uk/ego/DisplayGoTerm?id=GO:0006542) | 6 | 1.10E-06 |
| [cellular respiration](http://www.ebi.ac.uk/ego/DisplayGoTerm?id=GO:0045333) | 20 | 2.30E-06 |
| [tricarboxylic acid cycle](http://www.ebi.ac.uk/ego/DisplayGoTerm?id=GO:0006099) | 10 | 4.10E-06 |
| [acetyl-CoA catabolic process](http://www.ebi.ac.uk/ego/DisplayGoTerm?id=GO:0046356) | 10 | 4.10E-06 |
| [pyrimidine nucleotide biosynthetic process](http://www.ebi.ac.uk/ego/DisplayGoTerm?id=GO:0006221) | 8 | 5.70E-06 |
| [RNA processing](http://www.ebi.ac.uk/ego/DisplayGoTerm?id=GO:0006396) | 19 | 6.80E-06 |
| [amine biosynthetic process](http://www.ebi.ac.uk/ego/DisplayGoTerm?id=GO:0009309) | 25 | 8.10E-06 |
| [ncRNA processing](http://www.ebi.ac.uk/ego/DisplayGoTerm?id=GO:0034470) | 17 | 9.00E-06 |
| [cellular macromolecular complex disassembly](http://www.ebi.ac.uk/ego/DisplayGoTerm?id=GO:0034623) | 6 | 1.40E-05 |
| [macromolecular complex disassembly](http://www.ebi.ac.uk/ego/DisplayGoTerm?id=GO:0032984) | 6 | 1.40E-05 |
| [cellular component disassembly](http://www.ebi.ac.uk/ego/DisplayGoTerm?id=GO:0022411) | 6 | 1.40E-05 |
| [protein complex disassembly](http://www.ebi.ac.uk/ego/DisplayGoTerm?id=GO:0043241) | 6 | 1.40E-05 |
| [cellular protein complex disassembly](http://www.ebi.ac.uk/ego/DisplayGoTerm?id=GO:0043624) | 6 | 1.40E-05 |
| [cellular macromolecular complex subunit organization](http://www.ebi.ac.uk/ego/DisplayGoTerm?id=GO:0034621) | 10 | 1.40E-05 |
| [acetyl-CoA metabolic process](http://www.ebi.ac.uk/ego/DisplayGoTerm?id=GO:0006084) | 10 | 2.30E-05 |
| [ribonucleoside monophosphate biosynthetic process](http://www.ebi.ac.uk/ego/DisplayGoTerm?id=GO:0009156) | 8 | 2.30E-05 |
| [ribonucleoside monophosphate metabolic process](http://www.ebi.ac.uk/ego/DisplayGoTerm?id=GO:0009161) | 8 | 2.30E-05 |
| [amine catabolic process](http://www.ebi.ac.uk/ego/DisplayGoTerm?id=GO:0009310) | 13 | 3.00E-05 |
| [nucleoside triphosphate metabolic process](http://www.ebi.ac.uk/ego/DisplayGoTerm?id=GO:0009141) | 9 | 3.40E-05 |
| [nucleoside metabolic process](http://www.ebi.ac.uk/ego/DisplayGoTerm?id=GO:0009116) | 11 | 5.40E-05 |
| [nucleoside monophosphate metabolic process](http://www.ebi.ac.uk/ego/DisplayGoTerm?id=GO:0009123) | 8 | 7.10E-05 |
| [nucleoside monophosphate biosynthetic process](http://www.ebi.ac.uk/ego/DisplayGoTerm?id=GO:0009124) | 8 | 7.10E-05 |
| [tRNA processing](http://www.ebi.ac.uk/ego/DisplayGoTerm?id=GO:0008033) | 12 | 1.10E-04 |
| [RNA modification](http://www.ebi.ac.uk/ego/DisplayGoTerm?id=GO:0009451) | 12 | 1.20E-04 |
| [polyamine biosynthetic process](http://www.ebi.ac.uk/ego/DisplayGoTerm?id=GO:0006596) | 6 | 1.40E-04 |
| [energy coupled proton transport, down electrochemical gradient](http://www.ebi.ac.uk/ego/DisplayGoTerm?id=GO:0015985) | 7 | 1.50E-04 |
| [ATP synthesis coupled proton transport](http://www.ebi.ac.uk/ego/DisplayGoTerm?id=GO:0015986) | 7 | 1.50E-04 |
| [nucleoside triphosphate biosynthetic process](http://www.ebi.ac.uk/ego/DisplayGoTerm?id=GO:0009142) | 8 | 1.90E-04 |
| [aromatic compound biosynthetic process](http://www.ebi.ac.uk/ego/DisplayGoTerm?id=GO:0019438) | 12 | 2.20E-04 |
| [hydrogen transport](http://www.ebi.ac.uk/ego/DisplayGoTerm?id=GO:0006818) | 7 | 2.40E-04 |
| [proton transport](http://www.ebi.ac.uk/ego/DisplayGoTerm?id=GO:0015992) | 7 | 2.40E-04 |
| [pyrimidine base metabolic process](http://www.ebi.ac.uk/ego/DisplayGoTerm?id=GO:0006206) | 7 | 2.40E-04 |
| [purine ribonucleoside monophosphate biosynthetic process](http://www.ebi.ac.uk/ego/DisplayGoTerm?id=GO:0009168) | 6 | 2.40E-04 |
| [purine ribonucleoside monophosphate metabolic process](http://www.ebi.ac.uk/ego/DisplayGoTerm?id=GO:0009167) | 6 | 2.40E-04 |
| [purine nucleoside monophosphate metabolic process](http://www.ebi.ac.uk/ego/DisplayGoTerm?id=GO:0009126) | 6 | 2.40E-04 |
| [purine nucleoside monophosphate biosynthetic process](http://www.ebi.ac.uk/ego/DisplayGoTerm?id=GO:0009127) | 6 | 2.40E-04 |
| [glutamine family amino acid biosynthetic process](http://www.ebi.ac.uk/ego/DisplayGoTerm?id=GO:0009084) | 9 | 2.80E-04 |
| [heterocycle biosynthetic process](http://www.ebi.ac.uk/ego/DisplayGoTerm?id=GO:0018130) | 17 | 3.30E-04 |
| [nucleoside triphosphate biosynthetic process](http://www.ebi.ac.uk/ego/DisplayGoTerm?id=GO:0009142) | 8 | 1.90E-04 |
| [nucleobase biosynthetic process](http://www.ebi.ac.uk/ego/DisplayGoTerm?id=GO:0046112) | 7 | 3.70E-04 |
| [macromolecular complex subunit organization](http://www.ebi.ac.uk/ego/DisplayGoTerm?id=GO:0043933) | 10 | 3.80E-04 |
| [carboxylic acid biosynthetic process](http://www.ebi.ac.uk/ego/DisplayGoTerm?id=GO:0046394) | 23 | 4.60E-04 |
| [cellular amino acid biosynthetic process](http://www.ebi.ac.uk/ego/DisplayGoTerm?id=GO:0008652) | 20 | 4.80E-04 |
| [thiamin and derivative metabolic process](http://www.ebi.ac.uk/ego/DisplayGoTerm?id=GO:0042723) | 6 | 4.80E-04 |
| [thiamin biosynthetic process](http://www.ebi.ac.uk/ego/DisplayGoTerm?id=GO:0009228) | 6 | 4.80E-04 |
| [thiamin metabolic process](http://www.ebi.ac.uk/ego/DisplayGoTerm?id=GO:0006772) | 6 | 4.80E-04 |
| [thiamin and derivative biosynthetic process](http://www.ebi.ac.uk/ego/DisplayGoTerm?id=GO:0042724) | 6 | 4.80E-04 |
| [organic acid biosynthetic process](http://www.ebi.ac.uk/ego/DisplayGoTerm?id=GO:0016053) | 23 | 4.90E-04 |
| [amine transport](http://www.ebi.ac.uk/ego/DisplayGoTerm?id=GO:0015837) | 14 | 5.00E-04 |
| [pyrimidine base biosynthetic process](http://www.ebi.ac.uk/ego/DisplayGoTerm?id=GO:0019856) | 6 | 7.20E-04 |
| [biogenic amine biosynthetic process](http://www.ebi.ac.uk/ego/DisplayGoTerm?id=GO:0042401) | 7 | 8.50E-04 |
| [polyamine metabolic process](http://www.ebi.ac.uk/ego/DisplayGoTerm?id=GO:0006595) | 6 | 8.70E-04 |
| [ATP biosynthetic process](http://www.ebi.ac.uk/ego/DisplayGoTerm?id=GO:0006754) | 7 | 1.00E-03 |
| [ATP metabolic process](http://www.ebi.ac.uk/ego/DisplayGoTerm?id=GO:0046034) | 7 | 1.00E-03 |
| [purine nucleoside triphosphate biosynthetic process](http://www.ebi.ac.uk/ego/DisplayGoTerm?id=GO:0009145) | 7 | 1.10E-03 |
| [ribonucleoside triphosphate biosynthetic process](http://www.ebi.ac.uk/ego/DisplayGoTerm?id=GO:0009201) | 7 | 1.10E-03 |
| [purine ribonucleoside triphosphate biosynthetic process](http://www.ebi.ac.uk/ego/DisplayGoTerm?id=GO:0009206) | 7 | 1.10E-03 |
| [purine nucleoside triphosphate metabolic process](http://www.ebi.ac.uk/ego/DisplayGoTerm?id=GO:0009144) | 7 | 1.30E-03 |
| [purine ribonucleoside triphosphate metabolic process](http://www.ebi.ac.uk/ego/DisplayGoTerm?id=GO:0009205) | 7 | 1.30E-03 |
| [ribonucleoside triphosphate metabolic process](http://www.ebi.ac.uk/ego/DisplayGoTerm?id=GO:0009199) | 7 | 1.30E-03 |
| [glycine metabolic process](http://www.ebi.ac.uk/ego/DisplayGoTerm?id=GO:0006544) | 5 | 1.50E-03 |
| [biogenic amine metabolic process](http://www.ebi.ac.uk/ego/DisplayGoTerm?id=GO:0006576) | 9 | 1.50E-03 |
| [glutamine family amino acid metabolic process](http://www.ebi.ac.uk/ego/DisplayGoTerm?id=GO:0009064) | 12 | 1.70E-03 |
| [tRNA modification](http://www.ebi.ac.uk/ego/DisplayGoTerm?id=GO:0006400) | 6 | 1.70E-03 |
| [nucleobase metabolic process](http://www.ebi.ac.uk/ego/DisplayGoTerm?id=GO:0009112) | 8 | 1.80E-03 |
| ['de novo' pyrimidine base biosynthetic process](http://www.ebi.ac.uk/ego/DisplayGoTerm?id=GO:0006207) | 5 | 2.70E-03 |
| [transcription termination](http://www.ebi.ac.uk/ego/DisplayGoTerm?id=GO:0006353) | 3 | 3.10E-03 |
| [gluconeogenesis](http://www.ebi.ac.uk/ego/DisplayGoTerm?id=GO:0006094) | 5 | 3.50E-03 |
| [response to antibiotic](http://www.ebi.ac.uk/ego/DisplayGoTerm?id=GO:0046677) | 10 | 3.70E-03 |
| [hexose biosynthetic process](http://www.ebi.ac.uk/ego/DisplayGoTerm?id=GO:0019319) | 5 | 4.10E-03 |
| [ribonucleoside metabolic process](http://www.ebi.ac.uk/ego/DisplayGoTerm?id=GO:0009119) | 7 | 4.60E-03 |
| [ion transmembrane transport](http://www.ebi.ac.uk/ego/DisplayGoTerm?id=GO:0034220) | 7 | 4.60E-03 |
| [cellular amino acid derivative metabolic process](http://www.ebi.ac.uk/ego/DisplayGoTerm?id=GO:0006575) | 10 | 4.70E-03 |
| [oxidative phosphorylation](http://www.ebi.ac.uk/ego/DisplayGoTerm?id=GO:0006119) | 7 | 5.10E-03 |
| [monosaccharide biosynthetic process](http://www.ebi.ac.uk/ego/DisplayGoTerm?id=GO:0046364) | 6 | 5.10E-03 |
| [alcohol biosynthetic process](http://www.ebi.ac.uk/ego/DisplayGoTerm?id=GO:0046165) | 6 | 5.40E-03 |
| [tRNA aminoacylation for protein translation](http://www.ebi.ac.uk/ego/DisplayGoTerm?id=GO:0006418) | 6 | 6.00E-03 |
| [amino acid activation](http://www.ebi.ac.uk/ego/DisplayGoTerm?id=GO:0043038) | 6 | 6.00E-03 |
| [tRNA aminoacylation](http://www.ebi.ac.uk/ego/DisplayGoTerm?id=GO:0043039) | 6 | 6.00E-03 |
| [cellular amino acid derivative biosynthetic process](http://www.ebi.ac.uk/ego/DisplayGoTerm?id=GO:0042398) | 7 | 6.60E-03 |
| [phosphorus metabolic process](http://www.ebi.ac.uk/ego/DisplayGoTerm?id=GO:0006793) | 15 | 6.60E-03 |
| [pyruvate metabolic process](http://www.ebi.ac.uk/ego/DisplayGoTerm?id=GO:0006090) | 5 | 6.90E-03 |
| [glycerol metabolic process](http://www.ebi.ac.uk/ego/DisplayGoTerm?id=GO:0006071) | 5 | 6.90E-03 |
| [aspartate family amino acid metabolic process](http://www.ebi.ac.uk/ego/DisplayGoTerm?id=GO:0009066) | 7 | 7.10E-03 |
| [asparagine biosynthetic process](http://www.ebi.ac.uk/ego/DisplayGoTerm?id=GO:0006529) | 3 | 7.10E-03 |
| [water-soluble vitamin metabolic process](http://www.ebi.ac.uk/ego/DisplayGoTerm?id=GO:0006767) | 11 | 7.50E-03 |
| [IMP biosynthetic process](http://www.ebi.ac.uk/ego/DisplayGoTerm?id=GO:0006188) | 4 | 7.70E-03 |
| [IMP metabolic process](http://www.ebi.ac.uk/ego/DisplayGoTerm?id=GO:0046040) | 4 | 7.70E-03 |
| [amino acid transport](http://www.ebi.ac.uk/ego/DisplayGoTerm?id=GO:0006865) | 11 | 8.50E-03 |
| [purine ribonucleoside metabolic process](http://www.ebi.ac.uk/ego/DisplayGoTerm?id=GO:0046128) | 6 | 8.50E-03 |
| [anaerobic respiration](http://www.ebi.ac.uk/ego/DisplayGoTerm?id=GO:0009061) | 8 | 8.90E-03 |
| [purine nucleoside metabolic process](http://www.ebi.ac.uk/ego/DisplayGoTerm?id=GO:0042278) | 6 | 8.90E-03 |
| [coenzyme metabolic process](http://www.ebi.ac.uk/ego/DisplayGoTerm?id=GO:0006732) | 15 | 9.00E-03 |
| [water-soluble vitamin biosynthetic process](http://www.ebi.ac.uk/ego/DisplayGoTerm?id=GO:0042364) | 10 | 9.80E-03 |
| [regulation of cellular protein metabolic process](http://www.ebi.ac.uk/ego/DisplayGoTerm?id=GO:0032268) | 4 | 1.30E-02 |
| [regulation of translation](http://www.ebi.ac.uk/ego/DisplayGoTerm?id=GO:0006417) | 4 | 1.30E-02 |
| [serine family amino acid metabolic process](http://www.ebi.ac.uk/ego/DisplayGoTerm?id=GO:0009069) | 6 | 1.40E-02 |
| [vitamin metabolic process](http://www.ebi.ac.uk/ego/DisplayGoTerm?id=GO:0006766) | 11 | 1.70E-02 |
| [phosphate metabolic process](http://www.ebi.ac.uk/ego/DisplayGoTerm?id=GO:0006796) | 13 | 2.00E-02 |
| [vitamin biosynthetic process](http://www.ebi.ac.uk/ego/DisplayGoTerm?id=GO:0009110) | 10 | 2.20E-02 |
| [translational termination](http://www.ebi.ac.uk/ego/DisplayGoTerm?id=GO:0006415) | 3 | 2.20E-02 |
| [purine ribonucleoside biosynthetic process](http://www.ebi.ac.uk/ego/DisplayGoTerm?id=GO:0046129) | 4 | 2.50E-02 |
| [purine nucleoside biosynthetic process](http://www.ebi.ac.uk/ego/DisplayGoTerm?id=GO:0042451) | 4 | 2.50E-02 |
| [posttranscriptional regulation of gene expression](http://www.ebi.ac.uk/ego/DisplayGoTerm?id=GO:0010608) | 4 | 2.50E-02 |
| [ribonucleoside biosynthetic process](http://www.ebi.ac.uk/ego/DisplayGoTerm?id=GO:0042455) | 4 | 2.50E-02 |
| [nucleoside biosynthetic process](http://www.ebi.ac.uk/ego/DisplayGoTerm?id=GO:0009163) | 4 | 2.60E-02 |
| [dUTP metabolic process](http://www.ebi.ac.uk/ego/DisplayGoTerm?id=GO:0046080) | 2 | 2.90E-02 |
| [alditol metabolic process](http://www.ebi.ac.uk/ego/DisplayGoTerm?id=GO:0019400) | 5 | 3.20E-02 |
| [glycosaminoglycan biosynthetic process](http://www.ebi.ac.uk/ego/DisplayGoTerm?id=GO:0006024) | 5 | 3.30E-02 |
| [polyol metabolic process](http://www.ebi.ac.uk/ego/DisplayGoTerm?id=GO:0019751) | 5 | 3.30E-02 |
| [aminoglycan biosynthetic process](http://www.ebi.ac.uk/ego/DisplayGoTerm?id=GO:0006023) | 5 | 3.30E-02 |
| [peptidoglycan biosynthetic process](http://www.ebi.ac.uk/ego/DisplayGoTerm?id=GO:0009252) | 5 | 3.30E-02 |
| [polyamine transport](http://www.ebi.ac.uk/ego/DisplayGoTerm?id=GO:0015846) | 3 | 3.40E-02 |
| [glycine catabolic process](http://www.ebi.ac.uk/ego/DisplayGoTerm?id=GO:0006546) | 3 | 3.40E-02 |
| [serine family amino acid catabolic process](http://www.ebi.ac.uk/ego/DisplayGoTerm?id=GO:0009071) | 3 | 3.40E-02 |
| [xenobiotic metabolic process](http://www.ebi.ac.uk/ego/DisplayGoTerm?id=GO:0006805) | 4 | 3.50E-02 |
| [response to xenobiotic stimulus](http://www.ebi.ac.uk/ego/DisplayGoTerm?id=GO:0009410) | 4 | 3.50E-02 |
| [cell wall macromolecule biosynthetic process](http://www.ebi.ac.uk/ego/DisplayGoTerm?id=GO:0044038) | 5 | 3.70E-02 |
| [cellular component macromolecule biosynthetic process](http://www.ebi.ac.uk/ego/DisplayGoTerm?id=GO:0070589) | 5 | 3.70E-02 |
| [organic acid transport](http://www.ebi.ac.uk/ego/DisplayGoTerm?id=GO:0015849) | 12 | 3.80E-02 |
| [carboxylic acid transport](http://www.ebi.ac.uk/ego/DisplayGoTerm?id=GO:0046942) | 12 | 3.80E-02 |
| [regulation of cell shape](http://www.ebi.ac.uk/ego/DisplayGoTerm?id=GO:0008360) | 5 | 3.90E-02 |
| [cellular cell wall macromolecule metabolic process](http://www.ebi.ac.uk/ego/DisplayGoTerm?id=GO:0010382) | 5 | 3.90E-02 |
| [regulation of cell morphogenesis](http://www.ebi.ac.uk/ego/DisplayGoTerm?id=GO:0022604) | 5 | 3.90E-02 |
| [spermidine biosynthetic process](http://www.ebi.ac.uk/ego/DisplayGoTerm?id=GO:0008295) | 3 | 4.00E-02 |
| ['de novo' IMP biosynthetic process](http://www.ebi.ac.uk/ego/DisplayGoTerm?id=GO:0006189) | 3 | 4.00E-02 |
| [spermidine metabolic process](http://www.ebi.ac.uk/ego/DisplayGoTerm?id=GO:0008216) | 3 | 4.00E-02 |
| [aromatic amino acid family catabolic process](http://www.ebi.ac.uk/ego/DisplayGoTerm?id=GO:0009074) | 2 | 4.30E-02 |
| [pyrimidine salvage](http://www.ebi.ac.uk/ego/DisplayGoTerm?id=GO:0008655) | 2 | 4.30E-02 |
| [tryptophan catabolic process](http://www.ebi.ac.uk/ego/DisplayGoTerm?id=GO:0006569) | 2 | 4.30E-02 |
| [indolalkylamine catabolic process](http://www.ebi.ac.uk/ego/DisplayGoTerm?id=GO:0046218) | 2 | 4.30E-02 |
| [deoxyribonucleoside triphosphate metabolic process](http://www.ebi.ac.uk/ego/DisplayGoTerm?id=GO:0009200) | 2 | 4.30E-02 |
| [indole derivative catabolic process](http://www.ebi.ac.uk/ego/DisplayGoTerm?id=GO:0042436) | 2 | 4.30E-02 |
| [pyrimidine deoxyribonucleoside triphosphate metabolic process](http://www.ebi.ac.uk/ego/DisplayGoTerm?id=GO:0009211) | 2 | 4.30E-02 |
| [cofactor metabolic process](http://www.ebi.ac.uk/ego/DisplayGoTerm?id=GO:0051186) | 17 | 4.40E-02 |
| [pyrimidine nucleoside metabolic process](http://www.ebi.ac.uk/ego/DisplayGoTerm?id=GO:0006213) | 3 | 4.70E-02 |
| [cellular metabolic compound salvage](http://www.ebi.ac.uk/ego/DisplayGoTerm?id=GO:0043094) | 4 | 4.80E-02 |
| [peptidoglycan-based cell wall biogenesis](http://www.ebi.ac.uk/ego/DisplayGoTerm?id=GO:0009273) | 5 | 4.90E-02 |
| [cell wall biogenesis](http://www.ebi.ac.uk/ego/DisplayGoTerm?id=GO:0042546) | 5 | 4.90E-02 |
| [cellular amino acid catabolic process](http://www.ebi.ac.uk/ego/DisplayGoTerm?id=GO:0009063) | 6 | 4.90E-02 |
