# Supplementary material for: The Gluconeogenesis Pathway Is Involved in Maintenance of Enterohaemorrhagic Escherichia coli O157:H7 in Bovine Intestinal Content
Source: PLoS One. 2014 Jun 2;9(6):e98367. doi: 10.1371/journal.pone.0098367 (PMC4041753; doi:10.1371/journal.pone.0098367)
Supplement: Table S3 — Classification of genes down-regulated in EHEC EDL933 incubated in BSIC compared with cells incubated in M9-Glc. The genes were classified in different “Gene Ontology” (GO) categories with enrichment scores calculated for each genes group. (DOC) [file pone.0098367.s004.doc]

| **Table S3 Classification of genes down-regulated in EHEC EDL933 incubated in BSIC compared with cells incubated in M9-Glc.** | | |
| --- | --- | --- |
|  |  |  |
| GO category | Gene number | Enrichment score |
| [generation of precursor metabolites and energy](http://www.ebi.ac.uk/ego/DisplayGoTerm?id=GO:0006091) | 46 | 7.30E-21 |
| [anaerobic respiration](http://www.ebi.ac.uk/ego/DisplayGoTerm?id=GO:0009061) | 24 | 3.40E-19 |
| [energy derivation by oxidation of organic compounds](http://www.ebi.ac.uk/ego/DisplayGoTerm?id=GO:0015980) | 34 | 7.90E-19 |
| [carboxylic acid biosynthetic process](http://www.ebi.ac.uk/ego/DisplayGoTerm?id=GO:0046394) | 41 | 4.70E-18 |
| [organic acid biosynthetic process](http://www.ebi.ac.uk/ego/DisplayGoTerm?id=GO:0016053) | 41 | 5.50E-18 |
| [cellular amino acid biosynthetic process](http://www.ebi.ac.uk/ego/DisplayGoTerm?id=GO:0008652) | 36 | 5.50E-17 |
| [amine biosynthetic process](http://www.ebi.ac.uk/ego/DisplayGoTerm?id=GO:0009309) | 37 | 1.20E-16 |
| [sulfur metabolic process](http://www.ebi.ac.uk/ego/DisplayGoTerm?id=GO:0006790) | 26 | 3.00E-16 |
| [cellular respiration](http://www.ebi.ac.uk/ego/DisplayGoTerm?id=GO:0045333) | 27 | 9.90E-14 |
| [nitrogen compound biosynthetic process](http://www.ebi.ac.uk/ego/DisplayGoTerm?id=GO:0044271) | 46 | 1.60E-13 |
| [sulfur compound biosynthetic process](http://www.ebi.ac.uk/ego/DisplayGoTerm?id=GO:0044272) | 19 | 5.30E-12 |
| [sulfur amino acid biosynthetic process](http://www.ebi.ac.uk/ego/DisplayGoTerm?id=GO:0000097) | 12 | 6.50E-10 |
| [sulfur amino acid metabolic process](http://www.ebi.ac.uk/ego/DisplayGoTerm?id=GO:0000096) | 12 | 3.80E-09 |
| [cellular amino acid derivative biosynthetic process](http://www.ebi.ac.uk/ego/DisplayGoTerm?id=GO:0042398) | 10 | 5.80E-06 |
| [methionine biosynthetic process](http://www.ebi.ac.uk/ego/DisplayGoTerm?id=GO:0009086) | 7 | 7.20E-06 |
| [methionine metabolic process](http://www.ebi.ac.uk/ego/DisplayGoTerm?id=GO:0006555) | 7 | 7.20E-06 |
| [branched chain family amino acid biosynthetic process](http://www.ebi.ac.uk/ego/DisplayGoTerm?id=GO:0009082) | 8 | 2.40E-05 |
| [serine family amino acid biosynthetic process](http://www.ebi.ac.uk/ego/DisplayGoTerm?id=GO:0009070) | 7 | 5.80E-05 |
| [serine family amino acid metabolic process](http://www.ebi.ac.uk/ego/DisplayGoTerm?id=GO:0009069) | 8 | 1.20E-04 |
| [indolalkylamine biosynthetic process](http://www.ebi.ac.uk/ego/DisplayGoTerm?id=GO:0046219) | 5 | 1.40E-04 |
| [tryptophan biosynthetic process](http://www.ebi.ac.uk/ego/DisplayGoTerm?id=GO:0000162) | 5 | 1.40E-04 |
| [indole derivative biosynthetic process](http://www.ebi.ac.uk/ego/DisplayGoTerm?id=GO:0042435) | 5 | 1.40E-04 |
| [cellular amino acid derivative metabolic process](http://www.ebi.ac.uk/ego/DisplayGoTerm?id=GO:0006575) | 11 | 1.90E-04 |
| [tryptophan metabolic process](http://www.ebi.ac.uk/ego/DisplayGoTerm?id=GO:0006568) | 5 | 2.00E-04 |
| [indolalkylamine metabolic process](http://www.ebi.ac.uk/ego/DisplayGoTerm?id=GO:0006586) | 5 | 2.00E-04 |
| [indole derivative metabolic process](http://www.ebi.ac.uk/ego/DisplayGoTerm?id=GO:0042434) | 5 | 2.00E-04 |
| [indole and derivative metabolic process](http://www.ebi.ac.uk/ego/DisplayGoTerm?id=GO:0042430) | 5 | 2.00E-04 |
| [histidine biosynthetic process](http://www.ebi.ac.uk/ego/DisplayGoTerm?id=GO:0000105) | 6 | 2.50E-04 |
| [histidine family amino acid biosynthetic process](http://www.ebi.ac.uk/ego/DisplayGoTerm?id=GO:0009076) | 6 | 2.50E-04 |
| [branched chain family amino acid metabolic process](http://www.ebi.ac.uk/ego/DisplayGoTerm?id=GO:0009081) | 8 | 2.50E-04 |
| [glycolysis](http://www.ebi.ac.uk/ego/DisplayGoTerm?id=GO:0006096) | 8 | 2.70E-04 |
| [aspartate family amino acid biosynthetic process](http://www.ebi.ac.uk/ego/DisplayGoTerm?id=GO:0009067) | 8 | 3.10E-04 |
| [histidine family amino acid metabolic process](http://www.ebi.ac.uk/ego/DisplayGoTerm?id=GO:0009075) | 6 | 3.20E-04 |
| [histidine metabolic process](http://www.ebi.ac.uk/ego/DisplayGoTerm?id=GO:0006547) | 6 | 3.20E-04 |
| [aspartate family amino acid metabolic process](http://www.ebi.ac.uk/ego/DisplayGoTerm?id=GO:0009066) | 8 | 3.50E-04 |
| [S-adenosylmethionine biosynthetic process](http://www.ebi.ac.uk/ego/DisplayGoTerm?id=GO:0006556) | 3 | 3.80E-04 |
| [carbohydrate catabolic process](http://www.ebi.ac.uk/ego/DisplayGoTerm?id=GO:0016052) | 18 | 7.20E-04 |
| [glucose catabolic process](http://www.ebi.ac.uk/ego/DisplayGoTerm?id=GO:0006007) | 9 | 1.10E-03 |
| [hexose catabolic process](http://www.ebi.ac.uk/ego/DisplayGoTerm?id=GO:0019320) | 9 | 1.20E-03 |
| [biotin biosynthetic process](http://www.ebi.ac.uk/ego/DisplayGoTerm?id=GO:0009102) | 5 | 1.20E-03 |
| [biotin metabolic process](http://www.ebi.ac.uk/ego/DisplayGoTerm?id=GO:0006768) | 5 | 1.20E-03 |
| [amide biosynthetic process](http://www.ebi.ac.uk/ego/DisplayGoTerm?id=GO:0043604) | 5 | 1.20E-03 |
| [iron-sulfur cluster assembly](http://www.ebi.ac.uk/ego/DisplayGoTerm?id=GO:0016226) | 5 | 1.40E-03 |
| [metallo-sulfur cluster assembly](http://www.ebi.ac.uk/ego/DisplayGoTerm?id=GO:0031163) | 5 | 1.40E-03 |
| [biogenic amine biosynthetic process](http://www.ebi.ac.uk/ego/DisplayGoTerm?id=GO:0042401) | 6 | 1.70E-03 |
| [aromatic amino acid family biosynthetic process](http://www.ebi.ac.uk/ego/DisplayGoTerm?id=GO:0009073) | 6 | 1.70E-03 |
| [chorismate metabolic process](http://www.ebi.ac.uk/ego/DisplayGoTerm?id=GO:0046417) | 6 | 1.80E-03 |
| [aromatic amino acid family metabolic process](http://www.ebi.ac.uk/ego/DisplayGoTerm?id=GO:0009072) | 6 | 2.20E-03 |
| [glucose metabolic process](http://www.ebi.ac.uk/ego/DisplayGoTerm?id=GO:0006006) | 12 | 2.20E-03 |
| [monosaccharide catabolic process](http://www.ebi.ac.uk/ego/DisplayGoTerm?id=GO:0046365) | 9 | 2.30E-03 |
| [response to nutrient levels](http://www.ebi.ac.uk/ego/DisplayGoTerm?id=GO:0031667) | 5 | 2.60E-03 |
| [response to starvation](http://www.ebi.ac.uk/ego/DisplayGoTerm?id=GO:0042594) | 5 | 2.60E-03 |
| [dicarboxylic acid metabolic process](http://www.ebi.ac.uk/ego/DisplayGoTerm?id=GO:0043648) | 8 | 2.80E-03 |
| [cysteine biosynthetic process](http://www.ebi.ac.uk/ego/DisplayGoTerm?id=GO:0019344) | 4 | 3.10E-03 |
| [alcohol catabolic process](http://www.ebi.ac.uk/ego/DisplayGoTerm?id=GO:0046164) | 9 | 3.80E-03 |
| [homoserine metabolic process](http://www.ebi.ac.uk/ego/DisplayGoTerm?id=GO:0009092) | 3 | 4.30E-03 |
| [cofactor metabolic process](http://www.ebi.ac.uk/ego/DisplayGoTerm?id=GO:0051186) | 17 | 4.80E-03 |
| [cellular amide metabolic process](http://www.ebi.ac.uk/ego/DisplayGoTerm?id=GO:0043603) | 8 | 4.90E-03 |
| [cellular carbohydrate catabolic process](http://www.ebi.ac.uk/ego/DisplayGoTerm?id=GO:0044275) | 9 | 5.00E-03 |
| [water-soluble vitamin biosynthetic process](http://www.ebi.ac.uk/ego/DisplayGoTerm?id=GO:0042364) | 9 | 6.70E-03 |
| [biogenic amine metabolic process](http://www.ebi.ac.uk/ego/DisplayGoTerm?id=GO:0006576) | 7 | 6.80E-03 |
| [S-adenosylmethionine metabolic process](http://www.ebi.ac.uk/ego/DisplayGoTerm?id=GO:0046500) | 3 | 7.80E-03 |
| [cysteine metabolic process](http://www.ebi.ac.uk/ego/DisplayGoTerm?id=GO:0006534) | 4 | 9.70E-03 |
| [hexose metabolic process](http://www.ebi.ac.uk/ego/DisplayGoTerm?id=GO:0019318) | 12 | 1.30E-02 |
| [water-soluble vitamin metabolic process](http://www.ebi.ac.uk/ego/DisplayGoTerm?id=GO:0006767) | 9 | 1.30E-02 |
| [vitamin biosynthetic process](http://www.ebi.ac.uk/ego/DisplayGoTerm?id=GO:0009110) | 9 | 1.50E-02 |
| [amino acid transport](http://www.ebi.ac.uk/ego/DisplayGoTerm?id=GO:0006865) | 9 | 1.50E-02 |
| [amine transport](http://www.ebi.ac.uk/ego/DisplayGoTerm?id=GO:0015837) | 9 | 2.10E-02 |
| [vitamin metabolic process](http://www.ebi.ac.uk/ego/DisplayGoTerm?id=GO:0006766) | 9 | 2.60E-02 |
| [aromatic compound biosynthetic process](http://www.ebi.ac.uk/ego/DisplayGoTerm?id=GO:0019438) | 7 | 2.90E-02 |
| [cofactor biosynthetic process](http://www.ebi.ac.uk/ego/DisplayGoTerm?id=GO:0051188) | 11 | 3.30E-02 |
